# A gain-of-function mutation in ATP6V0A4 drives primary distal renal tubular alkalosis with enhanced V-ATPase activity

**DOI:** 10.1172/JCI188807

**Published:** 2025-04-29

**Authors:** Si-qi Peng, Qian-qian Wu, Wan-yi Wang, Yi-Lin Zhang, Rui-ning Zhou, Jun Liao, Jin-xuan Wei, Yan Yang, Wen Shi, Jun-lan Yang, Xiao-xu Wang, Zhi-yuan Wei, Jia-xuan Sun, Lu Huang, Hong Fan, Hui Cai, Cheng-kun Wang, Xin-hua Li, Ting-song Li, Bi-cheng Liu, Xiao-liang Zhang, Bin Wang

**Affiliations:** 1 Department of Nephrology, Zhong Da Hospital, Southeast University School of Medicine, Nanjing, China.; 2 Department of Physiology, School of Basic Medical Science, Nanjing Medical University, Nanjing, China.; 3 Nanjing Jiangbei New Area Biopharmaceutical Public Service Platform Co. Ltd, Nanjing, China.; 4 School of Science, China Pharmaceutical University, Jiangning District, Nanjing, China.; 5 Department of Medical Genetics and Developmental Biology, Medical School of Southeast University, The Key Laboratory of Developmental Genes and Human Diseases, Ministry of Education, Southeast University, Nanjing, China.; 6 Division of Renal Medicine, Department of Medicine, Emory University School of Medicine, Atlanta, Georgia, USA.; 7 Department of Infectious Diseases, Third Affiliated Hospital of Sun Yat-sen University, Guangzhou, China.; 8 Department of Neurology, Children’s Hospital of Chongqing Medical University, Chongqing, China.

**Keywords:** Genetics, Nephrology, Genetic diseases, Genetic variation

## Abstract

The *ATP6V0A4* gene encodes the a4 subunit of vacuolar H^+^-ATPase (V-ATPase), which mediates hydrogen ion transport across the membrane. Previous studies have suggested that mutations in *ATP6V0A4* consistently result in a loss of function, impairing the hydrogen ion transport efficacy of V-ATPase and leading to distal renal tubular acidosis and sensorineural hearing loss. Here, we identified a 32-year-old male patient and his father, both of whom harbored a heterozygous *ATP6V0A4* p.V512L mutation and exhibited hypochloremic metabolic alkalosis, acidic urine, and hypokalemia. Through a series of protein structural analyses and functional experiments, the V512L mutation was confirmed as a gain-of-function mutation in the *ATP6V0A4* gene. V512-a4 increased a4 subunit expression abundance by enhancing V512L-a4 stability and reducing its degradation, which in turn potentiated the capacity of V-ATPase to acidify the tubular lumen, leading to acidic urine and metabolic alkalosis. Through mutant V512L-a4 subunit structure-based virtual and experimental screening, we identified F351 (C_25_H_26_FN_3_O_2_S), a small-molecule inhibitor specifically targeting the V512L-a4 mutant. In conclusion, we identified a gain-of-function mutation in the *ATP6V0A4* gene, broadening its phenotypic and mutational spectrum, and we provide valuable insights into potential therapeutic approaches for diseases associated with *ATP6V0A4* mutations.

## Abstract 2

ATP6V0A4 p.V512 increased a4 subunit expression abundance by enhancing V512L-a4 stability and reducing its degradation, which in turn potentiated V-ATPase’s capacity to acidify the tubular lumen, leading to acidic urine and metabolic alkalosis.

## Graphical abstract

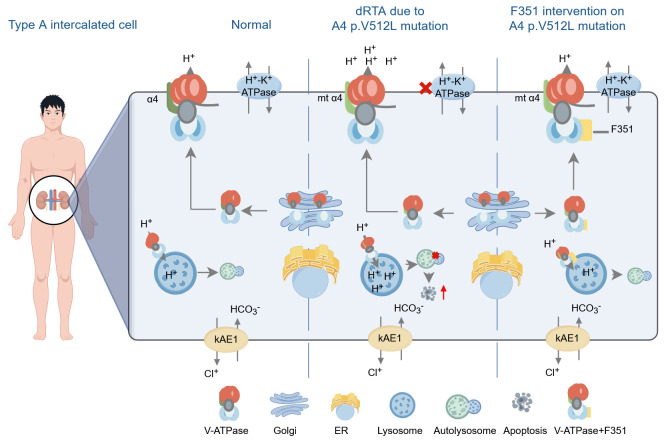

## Introduction

Vacuolar H^+^-ATPase (V-ATPase) is a membrane-anchored protein complex that mediates H^+^ transmembrane transport using energy obtained from ATP hydrolysis. It is primarily located in the plasma membrane and various intracellular membranes of eukaryotic cells and is widely expressed in human organs, including the kidney, testis, inner ear, bone, and skin (1, 2). V-ATPase has 2 structural domains, V1 and V0 (Supplemental Figure 1; supplemental material available online with this article; https://doi.org/10.1172/JCI188807DS1). V1 is located in the cytoplasm and is composed of 8 subunits (A_3_B_3_CDE_3_FG_3_H), primarily responsible for catalyzing the hydrolysis of ATP; V0 is anchored in the membrane and consists of a c-ring structure made up of 6 subunits (a, c, c″, d, e, RNAseK) and 2 subunits, ATP6AP1 and ATP6AP2, within the c-ring, mainly responsible for transmembrane transport of hydrogen ions (1). The V1 region couples with the V0 region via a rotating central rotor subcomplex. The peripheral stalk prevents the membrane-bound region from rotating with the rotor, forming the functional channel of V-ATPase (2). The proper assembly of the V1 and V0 structures is essential for the activity of V-ATPase (1).

Many subunits of V-ATPase exist in different isoforms, and the expression and localization of different isoforms of the same subunit are tissue specific and cell specific (Supplemental Table 1) (3). Notably, the B1 and a4 isoforms are highly and specifically expressed in the intercalated cells of the distal tubules and collecting ducts in the kidney (3). In renal type A intercalated cells, CO_2_ reacts with H_2_O to form H_2_CO_3_, catalyzed by carbonic anhydrase II (CA II). This H_2_CO_3_ then dissociates into H^+^ and HCO_3_^–^. The V-ATPase located on the luminal side of the cell membrane transports H^+^ into the tubular lumen, where it combines with NH_3_ to produce NH_4_^+^. Meanwhile, the Cl^–^/HCO_3_^–^ anion exchanger 1 (AE1) on the basolateral side of the membrane facilitates the transport of HCO_3_^–^ into the bloodstream (Supplemental Figure 2) (4). Mutations in the genes encoding these transporters impair the H^+^ transport function in type A intercalated cells. This impairment disrupts H^+^ secretion and reduces NH4^+^ secretion and ultimately decreases urine acidification (4).

In 1999, Karet et al. (5) first demonstrated that hereditary distal renal tubular acidosis (dRTA) is fully linked to the *ATP6V0A4* gene. This gene is located on chromosome 7q33-34 and encodes the a4 subunit of the V0 domain of V-ATPase, comprising 840 amino acids. It is specifically highly expressed in the kidney, epididymal, and inner ear tissues (3). The *ATP6V0A4* gene exhibits multiple types of mutations, including nonsense, frameshift, and splice mutations, which can severely disrupt protein structure. Nevertheless, missense mutations in the *ATP6V0A4* gene are pathogenic only if they alter amino acid residues in conserved subunit homologs (6, 7). In general, mutations of *ATP6V0A4* lead to loss-of-function (LOF) effects (4–6). Abnormalities in the a4 subunit, caused by these mutations, impair the assembly of V-ATPase, resulting in a complete or partial loss of V-ATPase activity. This dysfunction manifests as an inability of the kidneys to acidify the urine, leading to dRTA (4). Primary clinical characteristics of dRTA include hypokalemia, hyperchloremia, normal anion gap metabolic acidosis, paradoxically alkaline urine, and sensorineural hearing loss (4).

This study identified a heterozygous *ATP6V0A4* p.V512L mutation in a family with a clinical phenotype characterized by hypochloremic metabolic alkalosis, hypokalemia, and acidic urine. By integrating clinical phenotype evaluation, genetic variation analysis, molecular dynamics simulations, clinical samples, and a series of functional experiments, we confirmed that the *ATP6V0A4* p.V512L variant was a gain-of-function (GOF) mutation. V512L-a4 increased a4 subunit expression abundance by enhancing its stability and reducing degradation, which in turn potentiated V-ATPase’s capacity to acidify the tubular lumen, leading to acidic urine and metabolic alkalosis. A compensatory defect in H^+^-K^+^-ATPase activity reduced K^+^ reabsorption, ultimately resulting in hypokalemia. Furthermore, the V512L mutation caused lysosomal overacidification, resulting in impaired autophagic flux and increased apoptosis rates. More interestingly, through V512L-a4 subunit structure-based virtual screening, we discovered a small-molecule inhibitor, F351 (C_25_H_26_FN_3_O_2_S), that specifically targeted the V512L-a4 mutant. In vitro experiments verified that F351 effectively inhibited the V-ATPase activity of the mutant, reduced its H^+^ secretion capacity, and ultimately reversed the mutant phenotype.

## Results

### A patient with hypochloremic metabolic alkalosis, acidic urine, hypokalemia, and hearing loss carrying the ATP6V0A4 mutation p.V512L.

**Figure 1:**
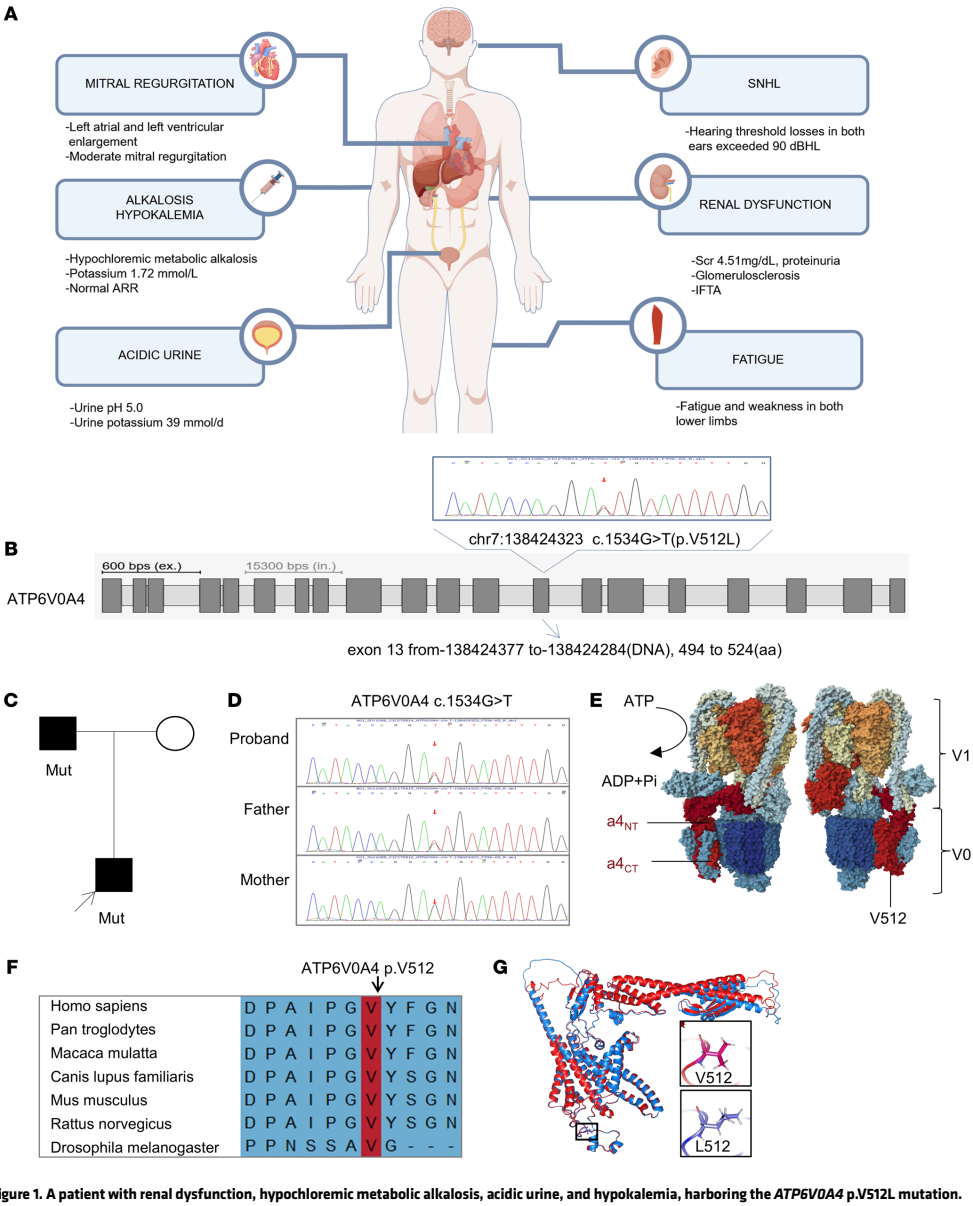
A patient with renal dysfunction, hypochloremic metabolic alkalosis, acidic urine, and hypokalemia, harboring the *ATP6V0A4* p.V512L mutation. (**A**) The clinicopathological characteristics of the patient. (**B**) Whole-exome sequencing identified a heterozygous mutation in the *ATP6V0A4* gene on chromosome 7 in the patient: c.1534G>T (p.Val512Leu). (**C** and **D**) Sanger sequencing of *ATP6V0A4*, amplified by PCR from leukocyte DNA of the patient and his parents, indicated that the father was a heterozygous carrier of the identified variant. (**E**) Cryo-EM density of human V-ATPase (PDB:7UNF) with subunits color-coded. Subunit a4 is labeled with red, and the N-terminal domain of the V-ATPase a4 subunit (a4-NT) is situated in the cytoplasm, interacting with the V1 domain to stabilize the enzyme structure; the C-terminal domain (a4-CT) forms a transmembrane proton channel. V512L residue is located at the C-terminus of the a4 subunit. (**F**) The valine residue altered by the p.V512L mutation is highly conserved across species. (**G**) Structure of WT-a4 and V512L-a4 as predicted by AlphaFold. SNHL, sensorineural hearing loss; ARR, aldosterone/renin ratio; IFTA, interstitial fibrosis and tubular atrophy; V512L, Val512Leu; Mut, mutant.

We investigated the *ATP6V0A4* genotype of a 32-year-old man presenting with hypochloremic metabolic alkalosis, acidic urine, hypokalemia, increased urinary excretion of potassium, and renal insufficiency (creatinine 4.51 mg/dL). The patient visited the clinic because of bilateral lower limb weakness, with other associated clinical features depicted in Figure 1A. Detailed laboratory findings are shown in Table 1. The patient’s blood pressure was 130/105 mmHg, and blood tests revealed normal levels of angiotensin II, renin, adrenaline, and noradrenaline (Supplemental Table 2), indicating a renal tubular acid-base imbalance resulting in increased urinary potassium excretion. Imaging showed normal bilateral kidneys, adrenal glands, and renal arteries (Supplemental Table 3). Echocardiography revealed left atrial and ventricular enlargement, and mitral regurgitation (Supplemental Table 3). The patient’s renal biopsy indicated severe tubular atrophy and interstitial fibrosis (Supplemental Figure 3). Subsequent auditory evaluations indicated that the patient had hearing threshold losses in both ears exceeding 80 decibel hearing level (dBHL), indicating sensorineural hearing loss. More importantly, despite adequate potassium supplementation, the patient continued to exhibit persistent acidic urine and alkalosis.

The patient’s father also had normal anion gap hypochloremic metabolic alkalosis, acidic urine, hypokalemia, and increased urinary potassium excretion (Table 1), but his phenotype was milder than that of the patient. Blood pressure, kidney function, and auditory tests for the patient’s father were within the normal range. In addition, the urine anion gap (UAG) value of the patient’s father was lower than that of normal individuals, which suggested that urinary ammonia excretion had likely increased (8, 9). Imaging revealed a single cyst in the right kidney, with no abnormalities in the remaining kidneys, adrenal glands, or renal vasculature (Supplemental Table 3). Echocardiography showed no abnormalities.

After whole-exome sequencing of the proband, we identified a heterozygous mutation in the *ATP6V0A4* gene on chromosome 7: c.1534G>T, p.Val512Leu (V512L) (Figure 1B), which to our knowledge had not been previously reported. Sanger sequencing revealed that the father was a *ATP6V0A4* p.V512L heterozygous carrier, whereas the mother did not carry this mutation, consistent with familial segregation (Figure 1, C and D). Sequence alignment showed that the Val512 residue was highly conserved across different species (Figure 1E). MutPred2, PANTHER, PolyPhen-2, and Mutation Taster determined that V512L was damaging, and the CADD prediction score was greater than 20, suggesting that V512L had a pathogenic effect (Supplemental Tables 4 and 5). Based on the above analysis, we considered *ATP6V0A4* p.V512L to be the pathogenic mutation in the proband and his father.

### Protein structure analysis and molecular dynamics simulations revealed conformational stability in the V512L-a4 subunit.

**Figure 2:**
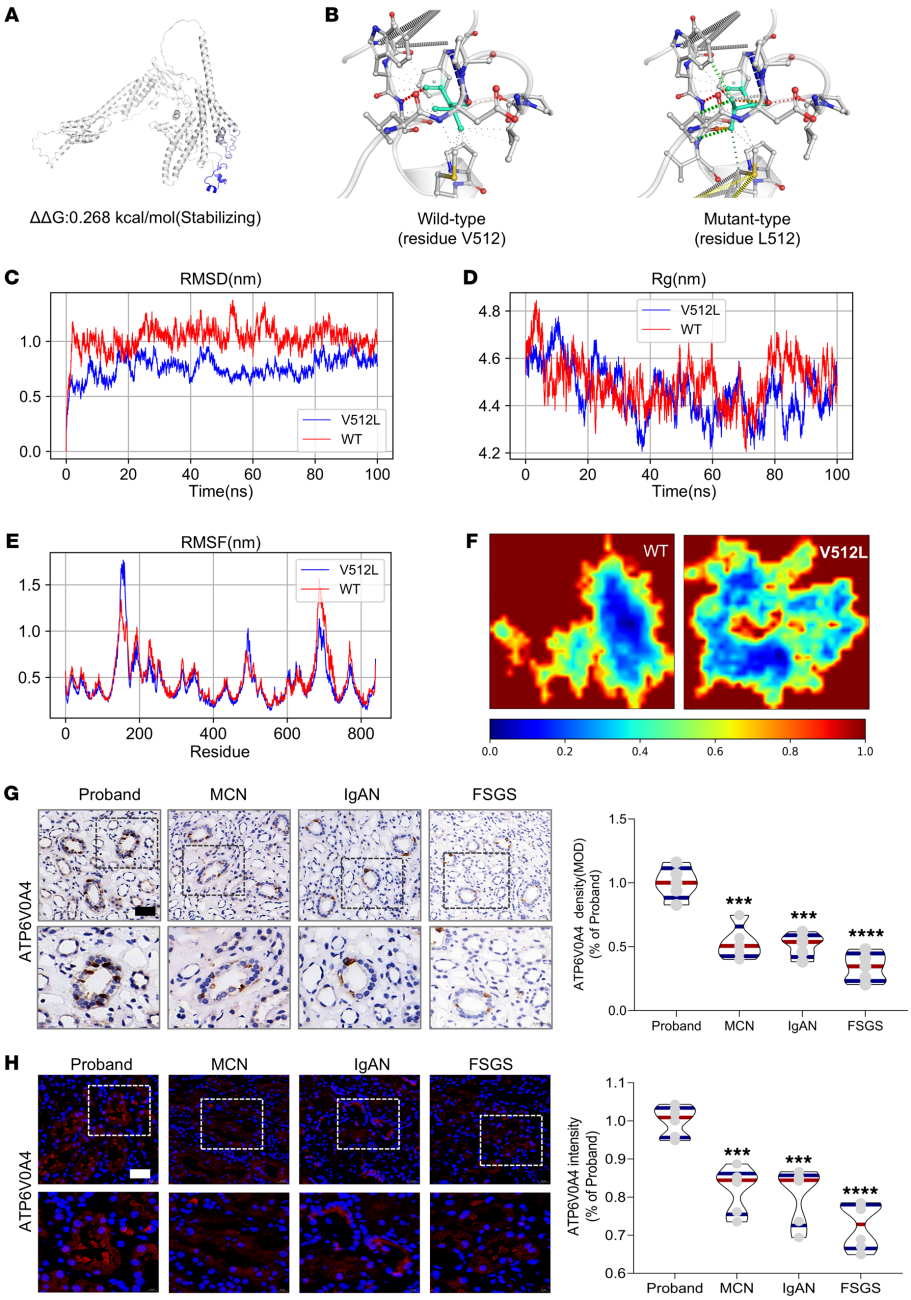
Molecular dynamics simulations and validation of patient kidney samples reveal V512L variant increases a4 protein stability and expression abundance. (**A**) The prediction outcome of V512L-a4 stability was ΔΔG:0.268 kcal/mol (stabilizing). (**B**) Prediction of interatomic interactions of p.V512L. Residues 512 in the WT-a4 and V512L-a4 proteins are colored in light green and are shown as sticks. The respective chemical interactions are labeled as dotted lines and colored as follows: hydrogen bonds—(red), weak hydrogen bonds—(orange), hydrophobic contacts—(green), amide-amide contacts—(blue), and ionic interactions—(gold). Amino acid residues are also colored according to type, namely: nitrogen (blue), oxygen (red), and sulfur (yellow). In comparison with WT sites, increased interactions were observed to be added in mutant sites. (**C**) The WT-a4 has an average RMSD of 1.03 nm, and the V512L-a4 is 0.76 nm. (**D**) The WT-a4 has an average Rg of 4.500 nm, and the V512L-a4 is 4.455 nm. (**E**) For the residues between 500 and 530 near the V512L, the WT-a4 has an average RMSF of 0.473 nm, and the V512L-a4 is 0.456 nm. (**F**) Free energy landscapes for WT-a4 and V512L-a4. The free energy landscape uses a color gradient from blue (indicating high stability, folded states) to red (indicating low stability, unfolded states). IHC (**G**) and immunofluorescence (**H**) validated patient renal tubular tissues with increased ATP6V0A4 expression abundance compared with MCN, IgAN, and FSGS. Each group was always compared with the proband, which was considered as the reference group. Scale bar: 40 μm in IHC and immunofluorescence. Violin plots indicate median (red) and upper and lower quartile (blue). ***P < 0.0005, ****P < 0.0001 by 2-tailed unpaired *t* test. V512L, Val512Leu; RMSD, root mean square deviation; Rg, radius of gyration; RMSF, root mean square fluctuations; MCN, minimal change nephropathy; IgAN, IgA nephropathy; FSGS, focal segmental glomerulosclerosis.

The proband who carried the *ATP6V0A4* p.V512L mutation exhibited hypochloremic metabolic alkalosis and acidic urine, which was contrary to the typical clinical phenotype of dRTA (4). We further investigated whether the *ATP6V0A4* p.V512L mutation resulted in increased V-ATPase activity and enhanced H^+^ secretion into the renal tubule lumen. The N-terminal domain of the V-ATPase a4 subunit (a4-NT) is situated in the cytoplasm, interacting with the V1 domain to stabilize the enzyme structure, and the C-terminal domain (a4-CT) forms a transmembrane proton channel (10) (Figure 1F). AlphaFold (11) predicted the highly accurate structure of the WT-a4 and V512L-a4 subunits (Supplemental Figure 4 and Supplemental Table 6) and identified V512L residue located at the C-terminus of the a4 subunit (Figure 1G), suggesting that V512L may affect transmembrane proton transport. The V512L mutation did not alter the electrostatic surface potential at the mutation site, with both WT-a4 and V512L-a4 subunits showing a neutral potential distribution (Supplemental Figure 5). This suggested that the V512L-a4 mutant achieved a more stable conformation, resulting in enhanced overall stability. We used DynaMut (http://biosig.unimelb.edu.au/dynamut/) to perform a thermodynamic stability analysis of V512L-a4 and WT-a4 proteins, revealing increased stability of V512L-a4 and enhanced intermolecular interactions near the V512L mutation site (Figure 2, A and B). This could be indicative of a potential association with GOF that could lead to increased production of active protein or reduced protein degradation.

A molecular dynamics simulation was performed over a 100 ns timescale, and plots of root mean square deviation (RMSD), root mean square fluctuations (RMSF), and radius of gyration (Rg) were generated to assess the flexibility and stability of the WT-a4 and V512L-a4 subunits. The V512L-a4 subunit had lower RMSD values than the WT-a4 subunit, suggesting that the V512L-a4 structure was more stable (Figure 2C). A low value of Rg for the V512L-a4 protein suggested more compact and tight protein structure (Figure 2D). A low value of RMSF for the V512L-a4 indicated that the mutant had marginally higher stability at the residue level (Figure 2E). Figure 2F shows that the V512L-a4 subunit had a greater prevalence of blue areas (indicating high stability, folded states) and a wider distribution compared with the WT-a4 subunit, suggesting increased stability in the mutant. Meanwhile, the secondary structure of V512L-a4 had no obvious changes compared with WT-a4 (Supplemental Figure 6 and Supplemental Table 7).

### ATP6V0A4 p.V512L mutation enhances stability of the V512L-a4 subunit and elevates V-ATPase activity.

**Figure 3:**
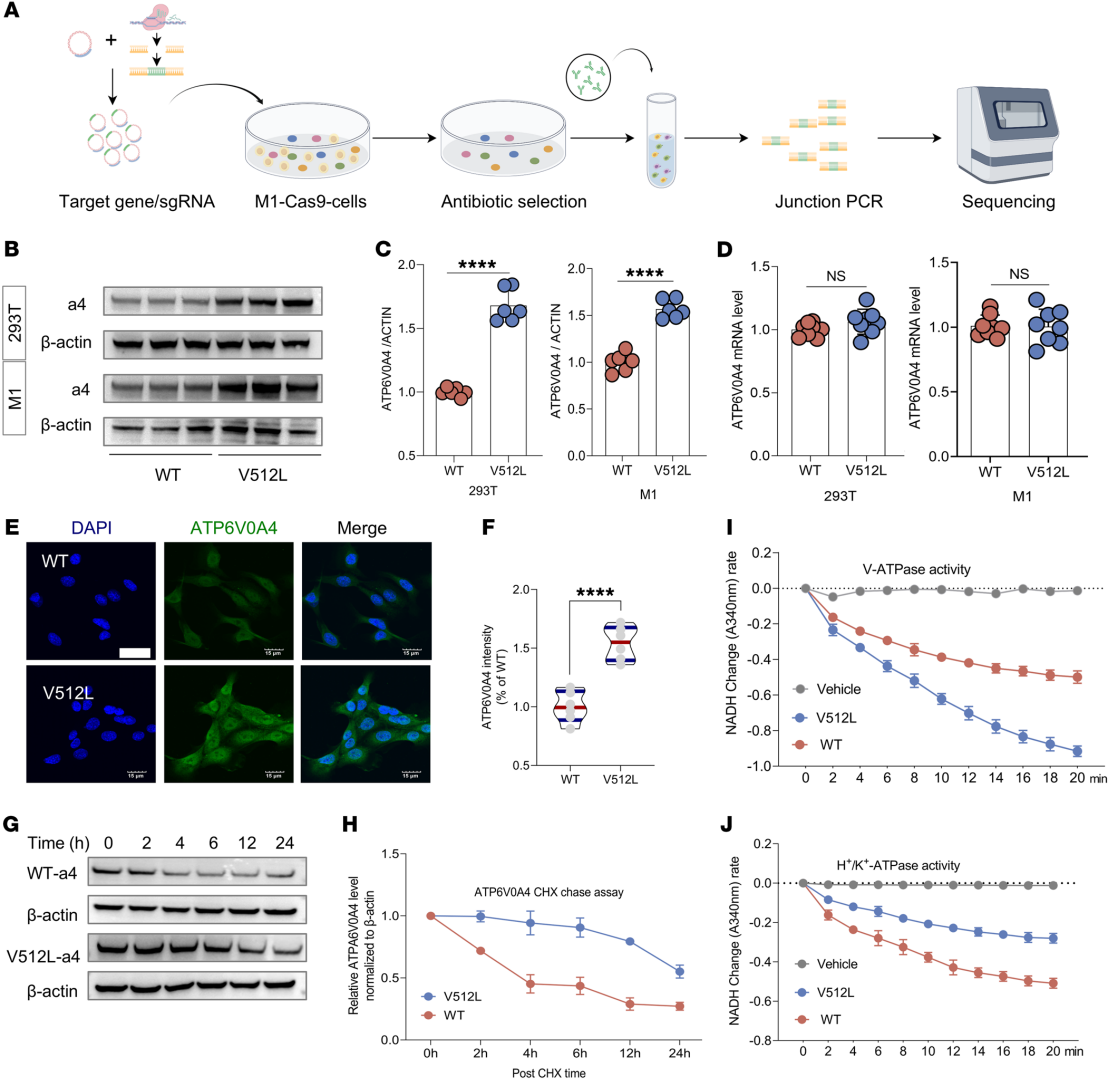
*ATP6V0A4* p.V512L mutation enhances stability of the a4 subunit and elevates V-ATPase activity. (**A**) The WT and V512L-transduced M1s were constructed using CRISPR/Cas9 genome editing. (**B** and **C**) Western blot and (**D**) RT-qPCR analysis of ATP6V0A4 expression abundance and mRNA levels in HEK293T cells and M1 cells expressing WT-a4 or V512L-a4. β-Actin antibody was used as a protein loading control. *n* = 6 per group (**C**); *n* = 8 per group (**D**). (**E** and **F**) Immunostaining results of ATP6V0A4 expression levels of WT and V512L-transduced M1s. Scale bar: 15 μm. Violin plots indicate median (red) and upper and lower quartile (blue). ****P < 0.0001 by 2-tailed unpaired *t* test. (**G** and **H**) CHX chasing experiment showing ATP6V0A4 stability. WT and V512L-transduced M1s were incubated with medium containing CHX (100 μg/mL). Cells were lysed at 0, 2, 4, 8, 12, and 24 hours after CHX treatment and subjected to Western blot. *n* = 3. (**I**) V-ATPase activity and (**J**) H^+^-K^+^-ATPase activity in M1 cells expressing WT-a4 or V512L-a4. *n* = 3. Data are given as mean ± SEM. M1, mouse collecting duct cells; a4, ATP6V0A4; CHX, cycloheximide.

Subsequently, we conducted in vitro experiments to determine whether the *ATP6V0A4* p.V512L mutation enhanced the stability and expression abundance of the a4 subunit. IHC and immunofluorescence analyses of renal biopsies from the proband and other patients indicated that the proband had higher renal tubular ATP6V0A4 protein expression abundance compared with patients with minimal change nephropathy, IgA nephropathy, or focal segmental glomerulosclerosis (Figure 2, G and H). In HEK293T cells, the V512L mutation led to a marked increase in the protein abundance of the a4 subunit, while *ATP6V0A4* mRNA levels remained unchanged (Figure 3, B–D). Next, we generated 2 stable mouse renal collecting duct cell lines (M1s) expressing WT-a4 and V512L-a4 using the CRISPR/Cas9 system (Figure 3A). Similarly, the V512L mutation increased the protein abundance of the a4 subunit in V512L-transduced M1s without affecting the corresponding mRNA levels (Figure 3, B–D). Immunofluorescence analysis of WT and V512L-transduced M1s also verified that the V512L mutation increased V512-a4 expression abundance (Figure 3, E and F). Cycloheximide (CHX) chase experiments showed that V512L-a4 proteins degraded more slowly than WT-a4 proteins, indicating increased stability, consistent with the molecular dynamics simulation results (Figure 3, G and H). We further verified that the V512L mutation noticeably elevated V-ATPase activity, leading to a compensatory decrease in H^+^-K^+^-ATPase activity (Figure 3, I and J). This finding aligns with previous animal studies, which showed that H^+^-K^+^-ATPase can partially compensate for V-ATPase function (12).

These results demonstrated that the V512L mutation enhanced V512L-a4 protein stability and reduced its degradation, which subsequently increased V512L-a4 expression abundance and elevated V-ATPase activity. Taken together, V512L was verified as a GOF mutation in the *ATP6V0A4* gene.

### ATP6V0A4 p.V512L mutation results in excessive lysosomal acidification and impaired lysosomal hydrolase activity.

**Figure 4:**
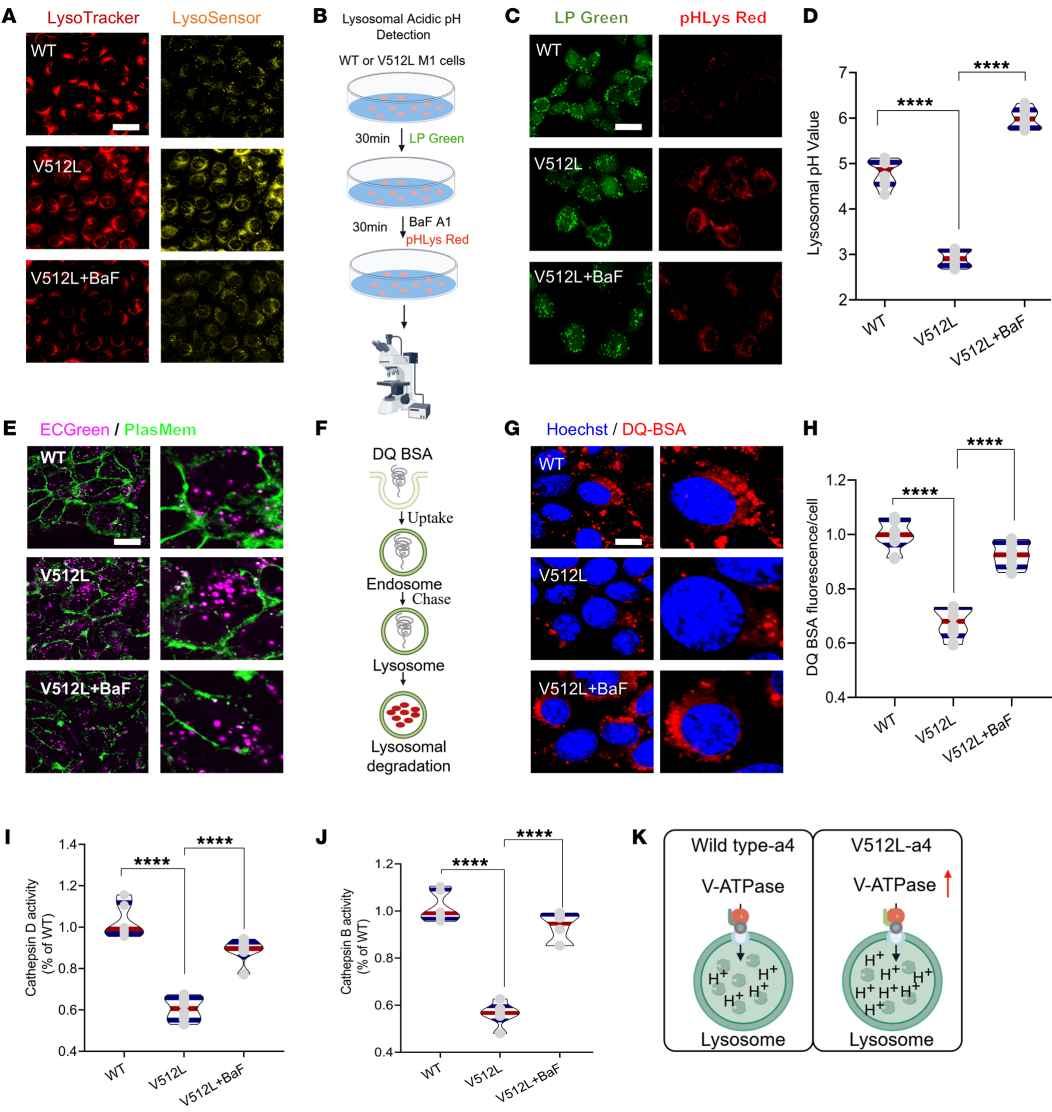
*ATP6V0A4* p.V512L mutation results in excessive lysosomal acidification and impaired lysosomal hydrolase activity. (**A**–**J**) BaF was applied to mildly (50 nm) block the V-ATPase to alkalize the lysosomes and endosomes of V512L-transduced M1s. (**A**) Lysosomal acidity assessed by LysoTracker (red) and LysoSensor (yellow) staining in WT and V512L-transduced M1s. BaF was applied to mildly (50 nm) block the V-ATPase to alkalize the lysosomes of V512L-transduced M1s. Scale bar: 30 μm. (**B** and **C**) Lysosomal pH in WT and V512L-transduced M1s determined using LysoPrime Green (green) and pHLys Red (red). Scale bar: 30 μm. (**D**) V512L mutation dropped the lysosomal pH from 5 to 2.5; BaF treatment significantly increased lysosomal pH to 6 in V512L-transduced M1s. *n* = 6 independent biology replicates. (**E**) Endosomal pH in WT and V512L-transduced M1s determined using ECGreen (purple). Cell membranes, the cell membranes were stained with PlasMem (green). Scale bar: 20 μm. (**F**) Lysosomal proteolytic activity was measured by DQ-BSA given that proteolysis of DQ-BSA releases protein fragments that are fluorescent. (**G**) Double immunostaining of DQ-BSA (red) and DAPI (blue) in WT and V512L-transduced M1s. Scale bar: 10 μm. (**H**) DQ-BSA staining was quantified by dividing total fluorescence intensity by the number of cells in each frame. *n* = 6. (**I**) Cathepsin D activity and (**J**) cathepsin B activity in WT and V512L-transduced M1s. *n* = 6. (**K**) V512L-a4 variant elevated the ability of the V-ATPase to acidify lysosomes, resulting in lysosomal hyperacidification and indirectly impairing lysosomal enzyme activity. Data are shown as mean ± SEM. Violin plots indicate median (red) and upper and lower quartile (blue). ****P < 0.0001 by 2-tailed unpaired *t* test. BaF, bafilomycin A1.

V-ATPase on the lysosomal membrane maintains pH homeostasis by transporting protons into the lysosomal lumen (13). Measurement of lysosomal pH with the cell-permeable dye LysoTracker and the pH-responsive dye LysoSensor showed that the *ATP6V0A4* p.V512L mutation markedly increased fluorescence intensity, suggesting hyperacidification of lysosomes (Figure 4A). Subsequently, V-ATPase inhibitor bafilomycin A1 reversed this phenomenon (Figure 4A). Similarly, the lysosomal acidic pH indicators pHLys Red and LysoPrime Green revealed a similar lysosomal hyperacidification in V512L-transduced M1s (Figure 4, B and C). The average lysosomal pH was 4.5 in WT and 3.0 in V512L-transduced M1s. Bafilomycin A1 treatment raised the lysosomal pH of mutant M1s to 5.5 (Figure 4D). V-ATPase pumps protons into endosomes, creating an acidic environment that promotes endocytosis and enhances ECGreen fluorescence. The *ATP6V0A4* p.V512L mutation was found to cause endosomal hyperacidification (Figure 4E).

The optimal pH for the proteolytic activity of the lysosomal enzyme is between 4.5 and 5.5 (14). Therefore, any disruption of lysosomal pH is likely to affect its proteolytic function. Lysosomal degradation activity was measured using dye-quenched red BSA (DQ red BSA). This substrate was internalized into cells via the endosomal pathway, and upon reaching the lysosome, it was cleaved by lysosomal hydrolases, releasing red fluorescent peptide fragments (Figure 4F). Microscopic images revealed decreased fluorescence intensity of DQ red BSA in V512L-transduced M1s, implying that the V512L mutation diminished lysosomal hydrolase activity. Treatment with bafilomycin A1 partially reversed the deficit in hydrolytic activity, although it did not restore activity to the levels observed in WT M1s (Figure 4, G and H). Cathepsin B (CTSB) and cathepsin D (CTSD) are abundant lysosomal proteases (15). V512L mutation substantially decreased CTSD and CTSB enzymatic activities (Figure 4, I and J). The findings suggested that the *ATP6V0A4* p.V512L GOF mutation enhanced the ability of V-ATPase to acidify lysosomes, leading to hyperacidification and indirectly impairing lysosomal enzyme proteolytic activity (Figure 4K).

### ATP6V0A4 p.V512L mutation blocked autophagic flux and promoted apoptosis.

**Figure 5:**
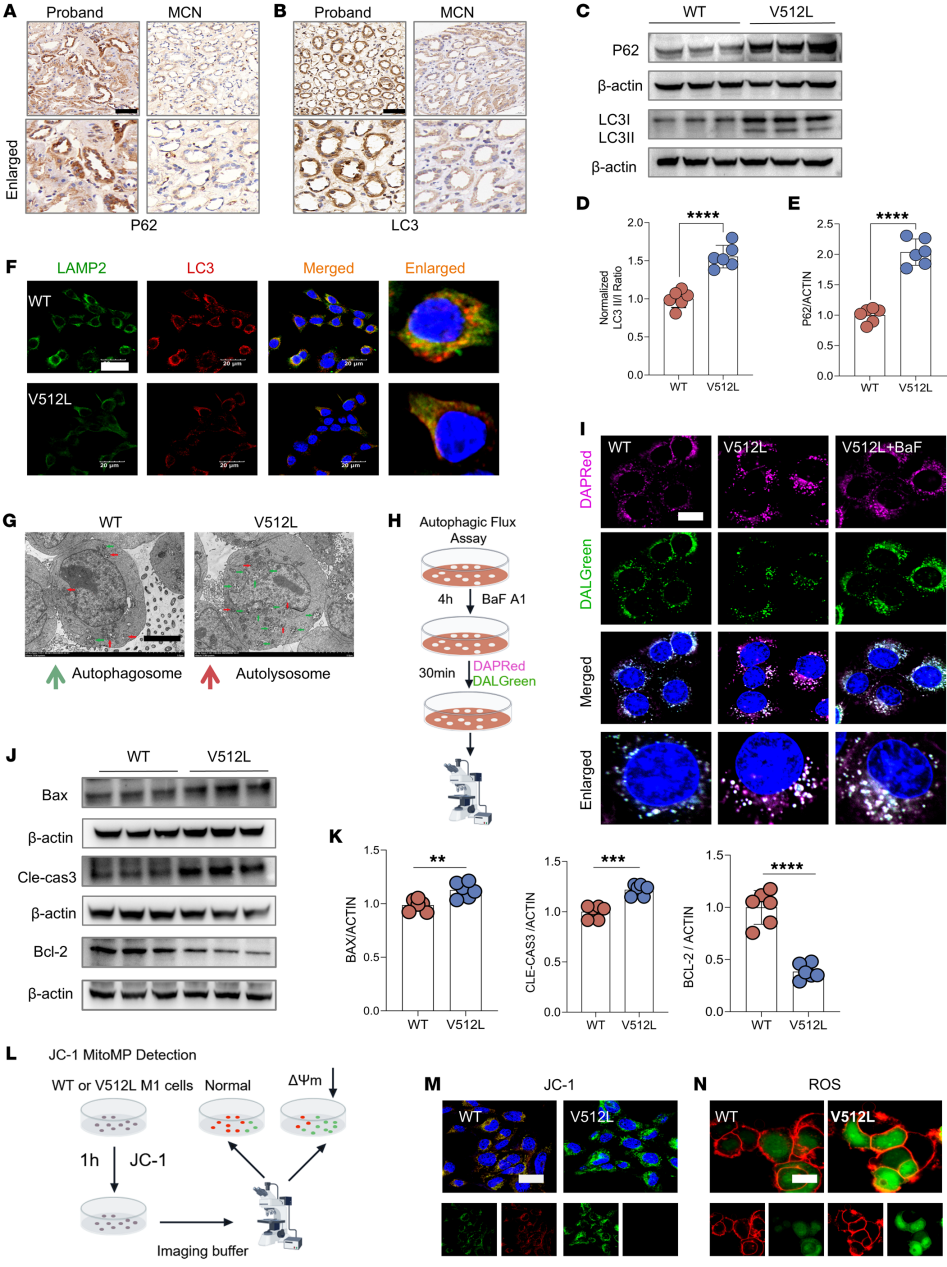
*ATP6V0A4* p.V512L mutation blocks autophagic flux and promotes apoptosis. (**A** and **B**) IHC verified the patient had increased renal tubular p62 and LC3 protein expression abundance compared with minimal change nephropathy. Scale bar: 40 μm. (**C**–**E**) Western blot of P62 and LC3 expression levels in M1 cells stably expressing WT-a4 and V512L-a4. β-Actin antibody was used as a protein loading control. *n* = 6. (**F**) V512L mutation attenuated the immunofluorescence colocalization of LC3 and LAMP2 proteins in M1 cells. Scale bar: 20 μm. (**G**) Compared with WT, transmission electron microscopy showed an increased number of autophagosomes in V512L-transduced M1s. Scale bar: 5 μm. (**H** and **I**) DAPRed and DALGreen fluorescent probes, used to monitor the formation of autophagosomes (purple) and autolysosomes (green), showed an accumulation of autophagosomes in V512L-transduced M1s. Bafilomycin A1 was applied to mildly (50 nm) block the V-ATPase to alkalize lysosomes of V512L-transduced M1s. Scale bar: 15 μm. (**J** and **K**) Western blot of bax, Cle-cas3, and bcl-2 expression levels in M1 cells stably expressing WT-a4 or V512L-a4, β-Actin antibody was used as a protein loading control. *n* = 6. (**L** and **M**) Mitochondrial membrane potential loss of V512L-transduced M1s was observed as a decrease in JC-1 red fluorescence and an increase in JC-1 green fluorescence. Nuclear, DAPI (blue). Scale bar: 40 μm. (**N**) Accumulation of ROS (green) was increased in V512L-transduced M1s. Cell membrane, PlasMem (red). Scale bar: 10 μm. Data are shown as mean ± SEM. **P < 0.005, ***P < 0.0005, ****P < 0.0001 by 2-tailed unpaired *t* test. Cle-cas3, cleaved caspase-3.

Lysosomes act as the terminal degradation centers in the autophagic process, and their dysfunction can lead to impaired autophagy (13). IHC results indicated that the proband had increased renal tubular p62 and LC3 protein expression abundance compared with a patient with minimal change nephropathy (Figure 5, A and B). Western blot analysis demonstrated that the V512L mutation considerably elevated LC3II/LC3I and P62 levels in V512L-transduced M1 cells (Figure 5, C–E). Colocalization analyses showed V512L mutation attenuated the colocalization between LC3 and LAMP2 in M1 cells, suggesting the blockage of the autophagosome-lysosome fusion process (Figure 5F). Transmission electron microscopy revealed a dramatic increase in the number of autophagosomes in V512L-transduced M1 cells, which did not appear to merge with lysosomes (Figure 5G). DAPRed and DALGreen fluorescent probes, designed to track the formation of autophagosomes (purple) and autolysosomes (green), demonstrated that autophagosomes accumulated in V512L-transduced M1s. Bafilomycin A1 treatment reduced the number of autophagosomes and increased the number of autolysosomes in V512L-transduced M1s (Figure 5, H and I). These results suggest that GOF mutation V512L jeopardized the function of lysosomes and reduced autophagosome-lysosome fusion, culminating in the blocking of autophagic flux.

Impaired autophagic clearance leads to the accumulation of inflammatory mediators and cell death products, often accompanied by ROS formation, ultimately promoting oxidative stress and apoptosis (16, 17). IHC results indicated that the proband had increased renal tubular bax protein expression abundance compared with a patient with minimal change nephropathy (Supplemental Figure 7). V512L mutation upregulated the expression of proapoptotic protein bax and cleaved caspase-3, and downregulated the antiapoptotic protein bcl-2, suggesting that the V512L mutation could facilitate apoptosis (Figure 5, J and K). Mitochondrial membrane potential loss of V512L-transduced M1s was observed as a decrease in JC-1 red fluorescence and an increase in JC-1 green fluorescence (Figure 5, L and M). Consistently, the V512L variant elicited a marked increase in intracellular ROS (Figure 5N).

### Protein structure–based virtual screening of targeted V512L-a4 mutant inhibitors.

**Figure 6:**
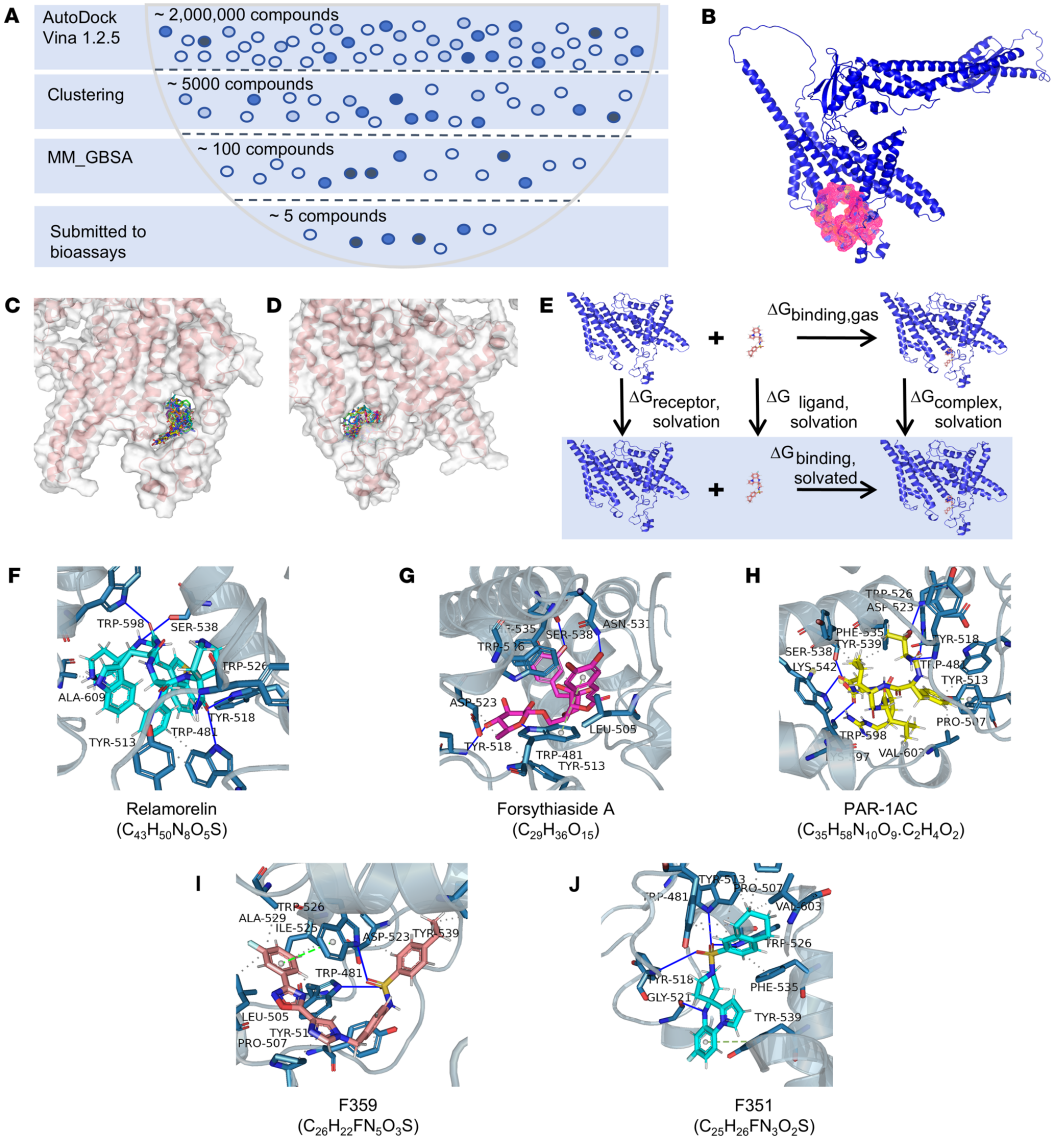
Protein structure–based virtual screening of targeted V512L-a4 inhibitors. (**A**) Structure-based virtual screening approach was performed in this study, and the workflow is shown. (**B**) The docking pockets of the V512L-a4 mutant subunit. The front (**C**) and rear (**D**) docking pose of mutant protein and 2 million ligand compounds. (**E**) MM-GBSA was used to predict the free energy of binding between the V512L-a4 mutant and the set of ligand compounds. (**F**–**J**) Five V512L-a4 mutant inhibitor compounds were selected: relamorelin, forsythiaside A, PAR-1AC, F359-0497 (F359), and F351-0364 (F351). The blue solid line represents hydrogen bonds, the gray dashed line represents hydrophobic interactions, and the green dashed line represents π-π interaction.

To generate the potential targeted inhibitors of the V512L-a4 mutant, a mutant protein structure–based virtual screening approach was performed in this study; the workflow is shown in Figure 6A. We employed the V512L-a4 mutant structure as the docking receptor and used CavityPlus (18) to predict the docking pockets of the target mutant protein through the incorporation of the positions of the mutant amino acid residue (Figure 6B). The docking pocket had a volume of 2674.38 Å³ and consisted of 12 H-bond donor centers, 9 H-bond acceptor centers, and 9 hydrophobic centers. CavityPlus predicted this docking pocket to have strong druggability. Figure 6, C and D, shows the front and rear docking pose of mutant protein and ligands. The screening for targeted V512L-a4 mutant inhibitors was conducted using 2 million compounds from the ChemDiv chemical library, the COCONUT natural product database (19), the MedChemExpress (MCE) active compound database, and the DrugBank database (20) (Figure 6A). Molecular docking analysis was performed using AutoDock Vina 1.2.5 to obtain docking scores (21), and the docked compounds were ranked based on docking scores (Figure 6A). Next, we selected the top 5,000 ranked compounds to perform clustering analysis based on structural similarity (22) (Figure 6A). We selected 100 representative compounds from the clusters for molecular dynamics simulations combined with thermodynamic analysis to calculate binding free energy (Figure 6, A and E).

We ultimately selected 5 targeted inhibitors of the V512L-a4 mutant: relamorelin; forsythiaside A; protease-activated receptor-1 agonist acetate (PAR-1AC); and 2 synthetic small molecules from ChemDiv, F359-0497 (F359) and F351-0364 (F351) (Figure 6, F–J). These compounds connected with the pocket through hydrogen bonds, hydrophobic interactions, and π-π interactions. All docking scores were below –10 kcal/mol, and MM-GBSA binding energies (23, 24) were below –40 kcal/mol, indicating strong interactions between the mutant protein and the targeted inhibitors. The structures, molecular formulas, and components of the binding free energy for these 5 compounds are presented in Supplemental Tables 8 and 9. All electrostatic interactions exhibited negative values, which may have contributed to the docking stability.

### F351 inhibits V-ATPase activity by targeting the V512L-a4 mutant in vitro.

**Figure 7:**
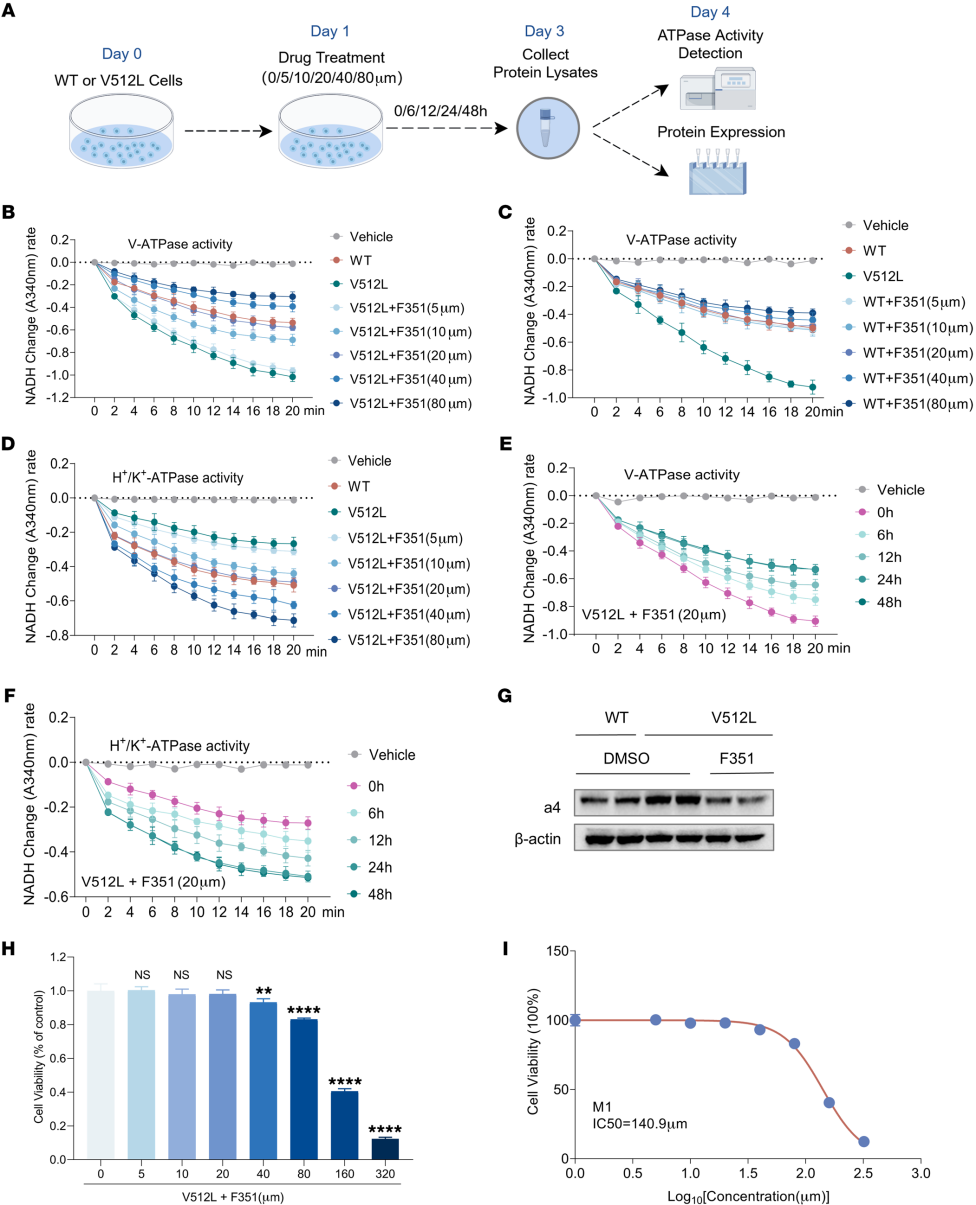
F351 inhibits V-ATPase activity by targeting the V512L-a4 mutant in vitro. (**A**) WT and V512L-transduced M1s were treated with indicated concentrations of selected small molecule compounds and were collected at each indicated time point for analysis. F351 was obtained as a white amorphous powder with molecular formula C_25_H_26_FN_3_O_2_S and a molecular weight of 451.6 g/mol. (**B**) F351 inhibited V-ATPase activity of V512L-a4 mutant in a concentration-dependent manner. *n* =3. (**C**) F351 had no stable inhibitory effect on the V-ATPase activity of WT. *n* = 3. (**D**) F351 increased H^+^-K^+^-ATPase activity of V512L-a4 mutant in a concentration-dependent manner. *n* = 3. At a drug concentration of 20 μmol/L, V-ATPase activity (**B**) and H^+^-K^+^-ATPase activity (**D**) of V512L-transduced M1s were restored to a level equivalent to that of WT. (**E** and **F**) Based on the optimal concentration 20 μmol/L, we found that the ideal time point for F351 interventions was 24 hours. *n* = 3. (**G**) V512L-transduced M1s were treated with F351 at an optimal concentration of 20 μmol/L for 24 hours, resulting in significant downregulation of V512L-a4 abundance. DMSO was added as solvent control. (**H** and **I**) CCK8 screening revealed that 50% cell death of V512L-transduced M1s occurred only at F351 concentrations exceeding 140.9 μmol/L, indicating low cytotoxicity. *n* = 6. Each group was always compared with the first group, which was considered as the reference group. Data are shown as mean ± SEM. **P < 0.005, ****P < 0.0001 by 2-tailed unpaired *t* test. F351, F351-0364.

To further evaluate the potential of these 5 compounds as inhibitors of the V512L-a4 mutant, we conducted V-ATPase activity assays and ATP6V0A4 (a4) expression assays in WT and V512L-transduced M1s after treatment with tested compounds (Figure 7A). After 24 hours of incubation, no pronounced, stable inhibition of V-ATPase activity was observed in V512L-transduced M1s after exposure to different concentrations of relamorelin, forsythiaside A, PAR-1AC, and F359 (Supplemental Figure 8, A–D). The most stable and effective compound for inhibiting V-ATPase activity in V512L-transduced M1s was F351 (Figure 7B). At a drug concentration of 20 μmol/L, V-ATPase activity was restored to a level equivalent to that of WT-a4 M1s, and the drug effect was concentration dependent. F351 was obtained as a white amorphous powder with the molecular formula C_25_H_26_FN_3_O_2_S and a molecular weight of 451.6 g/mol (Supplemental Table 8). Its simple molecular structure contributed to its high drug-likeness, enhancing its potential as a therapeutic agent. Additionally, F351 treatment in WT-a4 M1s did not result in marked inhibition of intracellular V-ATPase activity, suggesting that F351 specifically targeted and inhibited V-ATPase activity in V512L-transduced M1s (Figure 7C).

**Figure 8:**
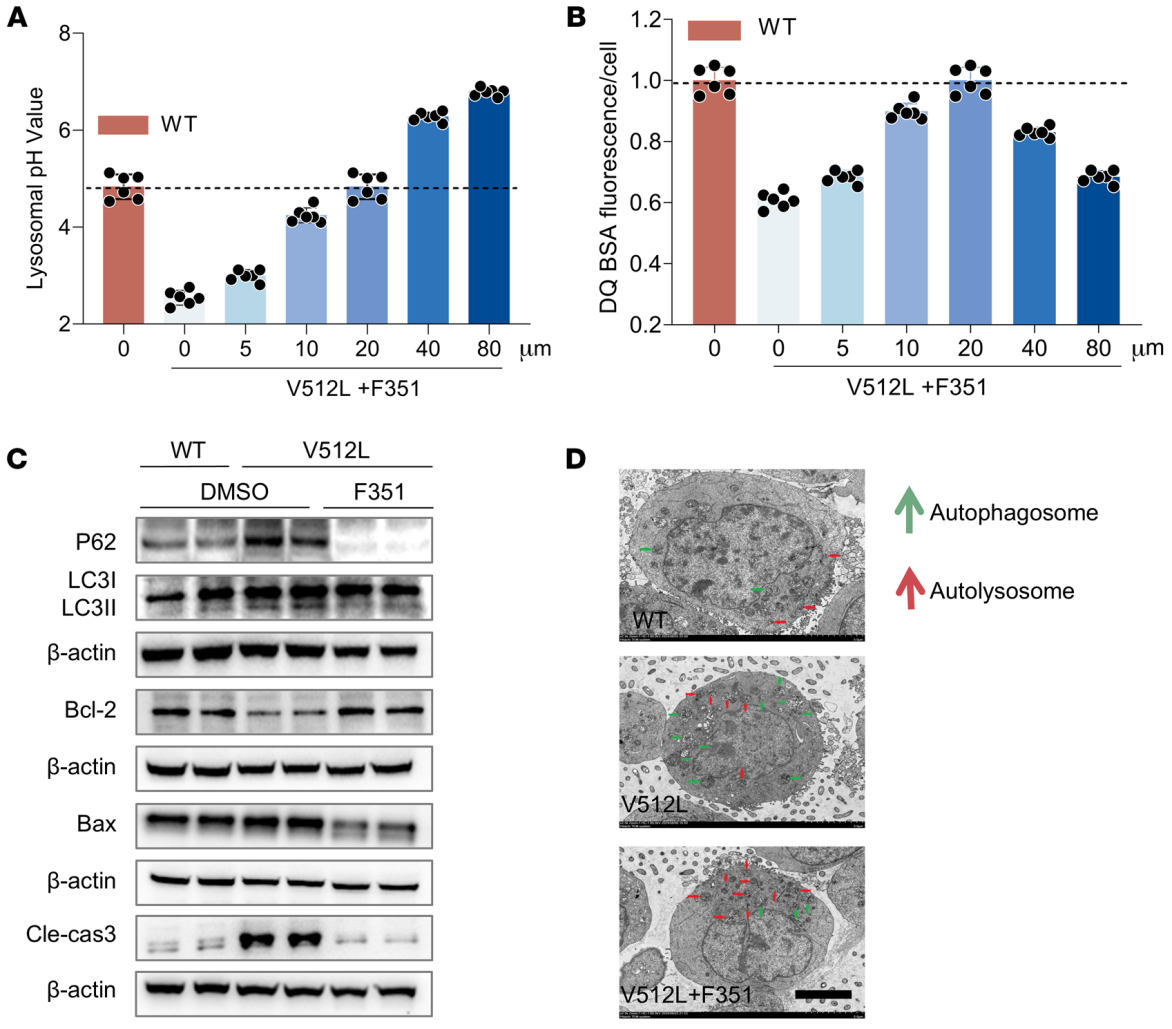
F351 elevates lysosomal pH, promotes autophagosome-lysosome fusion, and mitigates apoptosis by targeting the V512L-a4 mutant in vitro. F351 elevated lysosomal pH (**A**) and lysosomal degradation activity (**B**) of the V512L-a4 mutant in a concentration-dependent manner. At a drug concentration of 20 μmol/L, lysosomal degradation activity was restored to a level equivalent to that of WT. All data are shown as mean ± SEM, *n* = 6. (**C**) F351 markedly reversed the expression of autophagy (P62, LC3) and apoptosis-related (bax, bcl-2, cleaved caspase-3) proteins in the V512L-a4 mutant. DMSO was added as solvent control. (**D**) F351 promoted the fusion of autophagosomes and lysosomes in the V512L-a4 mutant. Double membrane autophagosomes (green), single membrane autolysosomes (red). Scale bars: 5 μm.

Next, we observed a compensatory increase in H^+^-K^+^-ATPase activity in V512L-transduced M1s treated with F351, with the most pronounced effect at a concentration of 20 μmol/L (Figure 7D). Based on the optimal concentrations, we found that the ideal time point for further interventions was 24 hours (Figure 7, E and F). Treating V512L-transduced M1s with F351 at an optimal concentration of 20 μmol/L for 24 hours led to a noticeable reduction in a4 expression abundance (Figure 7G). CCK8 screening showed that 50% cell death occurred only at F351 concentrations above 140.9 μmol/L, and the optimal effective concentration of F351 was 20 μmol/L, indicating low cytotoxicity (Figure 7, H and I). Subsequently, in HEK293T cells, the inhibitory effect of F351 on the V512L-a4 mutant was consistent with that observed in M1 cells (Supplemental Figure 9, A–D). Finally, F351 treatment resulted in a concentration-dependent increase in lysosomal pH and degradative activity in V512L-transduced M1s (Figure 8, A and B). At a concentration of 20 μmol/L, the lysosomal pH was closest to that of WT M1 cells, with optimal degradation activity. Moreover, F351 markedly reversed the expression of proteins related to autophagy and apoptosis (Figure 8C) and promoted the fusion of autophagosomes and lysosomes in V512L-transduced M1s (Figure 8D). Taken together, these data demonstrated that F351 could be a potential candidate for treating tubular acid-base imbalance caused by the *ATP6V0A4* p.V512L mutation and may serve as a lead compound for further optimization.

## Discussion

Here, we report a family diagnosed with hypochloremic metabolic alkalosis, acidic urine, and hypokalemia, who carry a missense variant c.1534G>T (p.Val512Leu) in the *ATP6V0A4* gene. The V512L variant increased a4 subunit expression abundance by enhancing its stability and reducing degradation. This elevated the ability of V-ATPase to acidify the tubular lumen, leading to acidic urine and metabolic alkalosis. On the basis of the mechanistic findings, we discovered a small-molecule inhibitor, F351 (C_25_H_26_FN_3_O_2_S), that targeted the V512L-a4 mutant through structure-based virtual screening. F351 effectively reduced the expression abundance of the a4 subunit; attenuated the hydrogen-secreting ability of V-ATPase; and reversed V512L-induced lysosomal hyperacidification, autophagy blockade, and apoptosis activation.

The V-ATPase is a tightly coupled enzyme that exhibits activity only when the cytosolic V1 domain and transmembrane V0 domain are correctly assembled (1). Subunit a comprises 4 isoforms (a1–a4) and is located in the V0 domain, which is responsible for the subcellular localization of V-ATPase and the formation of the transmembrane proton channel (3, 25). Previous studies have confirmed that all *ATP6V0A4* pathogenic variants described to date are LOF mutations (4, 5). Structural abnormalities in the a4 subunit, caused by *ATP6V0A4* pathogenic mutations, can decrease the protein expression abundance and hinder the proper assembly of V-ATPase, thereby reducing the ability of type A intercalated cells to pump H^+^ into the lumen, which ultimately leads to the occurrence of autosomal recessive inheritance (AR) dRTA (4, 5, 26). Protein structure analysis and molecular dynamics simulations demonstrated that the V512L GOF mutation increased the stability and compactness of the a4 protein, potentially facilitating the assembly of the V0 and V1 domains. Subsequent in vitro functional experiments confirmed that V512-a4 had a slower degradation rate and higher expression abundance, enhancing V-ATPase activity both qualitatively and quantitatively.

Moreover, Kawasaki-Nishi et al. (27) indicated that the N-terminal domain of the V-ATPase a subunit regulates targeting and in vivo dissociation, while the C-terminal domain influences coupling efficiencies, particularly in the energy conversion between proton transport and ATP hydrolysis. Interestingly, subsequent studies further revealed that mutations in the nonhomologous domains of the d and A subunits in yeast cells improved V-ATPase coupling efficiency and increased proton transport, suggesting that the coupling efficiency of WT V-ATPase may not be optimal (28, 29). However, no comparative studies have been conducted to investigate the coupling efficiency of V-ATPase bearing the WT and mutant subunit a. The GOF mutation V512L identified in our study, located on the C-terminal of V-ATPase, may enhance coupling efficiency, thereby improving the energy conversion of ATP hydrolysis and further promoting proton transport. However, this needs to be confirmed through further detailed studies in basic cell biology.

The proband’s clinical signs included hypochloremic metabolic alkalosis, acidic urine, hypokalemia, renal dysfunction, hypertension, left atrial and ventricular enlargement, mitral regurgitation, and sensorineural hearing loss. Acidic urine and alkalosis are both attributed to the increased activity of V-ATPase, which transports H^+^ into the tubular lumen, where it combines with NH3 to produce NH4^+^ (4). Consequently, an increase in V-ATPase activity is theoretically associated with enhanced urinary ammonium excretion. UAG calculation serves as an approximate indicator of urinary ammonium during the initial bedside assessment of metabolic acidosis (8, 9). In dRTA, impaired hydrogen ion secretion reduces urinary NH4^+^ excretion and elevates urinary UAG (8). In chronic kidney disease, ammonium excretion capacity is significantly reduced, with declining kidney function (8), leading to a lack of correlation between V-ATPase activity and urinary ammonia excretion. Moreover, in chronic kidney disease, UAG has exhibited a weak and direct correlation with ammonia excretion (30). In this study, urinary NH4^+^ was not measured, but urinary UAG was assessed. The proband’s father, with a normal glomerular filtration rate, exhibited a negative UAG, suggestive of increased urinary ammonium excretion.

Hypokalemia may result from renal potassium loss and a shift of potassium from extracellular to intracellular. Excessive hydrogen secretion by type A intercalated cells caused a negative intracellular hydrogen ion balance, triggering a compensatory decrease in H^+^-K^+^-ATPase exchange and ultimately resulting in hypokalemia (31). Additionally, persistent metabolic alkalosis promotes the shift of potassium from the extracellular to the intracellular compartment (32), exacerbating hypokalemia. However, animal experiments have confirmed that H^+^-K^+^-ATPase cannot adequately compensate for the function of V-ATPase, contributes minimally to pH homeostasis in the human kidney, and cannot reverse renal pH imbalance (12). Hypokalemia induces depolarization of the resting membrane potential, thereby reducing neuromuscular excitability and leading to muscle weakness (33). However, muscle paralysis is rare, as nerve conduction is typically preserved. After potassium supplementation, the patient’s serum potassium levels normalized, and bilateral lower limb weakness gradually improved. However, the urinary pH remained at 5 or less, indicating persistent acidic urine. This further supports the notion that the acidic urine was not caused by hypokalemia, but rather by increased hydrogen ion secretion from the kidneys. Hypochloremia may occur secondary to increased Cl^–^-HCO3^–^ exchange on the basolateral side of type A intercalated cells, diminished Cl^–^ reabsorption in renal tubules, and Cl^–^ transfer from extracellular to intracellular spaces (4, 34).

Patients with dRTA usually present with normal or near normal glomerular filtration rate, but slow progression to chronic kidney disease has been documented (35). Hypokalemia may serve as a key contributor to the progression of chronic kidney disease in this patient (36). Chronic potassium deficiency induces vacuolar degeneration of renal tubular epithelial cells, interstitial inflammation, and fibrosis (36, 37). Early animal studies, induced by dietary potassium depletion, showed that sustained hypokalemia (2–12 weeks) led to tubular epithelial cell swelling, vacuolation, fatty degeneration, and calcification, as well as thickening and fibrosis of the thick ascending limb’s basement membrane, accompanied by necrosis and atrophy (38, 39). This suggests that chronic hypokalemia leads to a progressive deterioration of renal function, ultimately advancing to chronic kidney disease. Hypertension, left atrial and ventricular enlargement, and mitral regurgitation are likely sequelae of renal dysfunction (40), rather than direct outcomes of genetic mutations. The milder phenotypes of acidic urine, alkalosis, and hypokalemia in the patient’s father indicate a less severe renal acid-base imbalance, which facilitates enhanced renal compensation and minimizes potassium suppression. Consequently, the father did not exhibit muscle weakness, renal insufficiency, or cardiac damage typically associated with chronic kidney failure. Furthermore, the *ATP6V0A4* variant disrupts inner ear pH balance, gradually reducing hair cell sensitivity and causing a progressive decline, mainly presenting as late-onset sensorineural hearing loss (4).

The optimal activity of lysosomal hydrolases occurs at a pH of 4.5–5.0, and this acidic environment is maintained by V-ATPase–mediated proton transport on the lysosomal membrane (14, 41). The V512L-a4 GOF mutation promotes V-ATPase activation, increases hydrogen ion release, and leads to excessive lysosomal acidification and impaired lysosomal hydrolytic function. Lysosomal dysfunction is known to subvert autophagy, resulting in the accumulation of autophagosomes/autolysosomes and their engulfed materials due to the lack of the final recycling step (13). Extensive research has established that autophagy activation in renal tubular cells acts as a protective mechanism in both acute and chronic kidney diseases. Conversely, dysregulated autophagy is a major cause of renal cell injury (16). The V512L-a4 GOF mutation causes lysosomal dysfunction, leading to disrupted autophagosome-lysosome fusion and triggering a blockage in autophagic flux. Impaired autophagy leads to a reduced capacity for cellular clearance, often accompanied by increased ROS production, which subsequently induces heightened oxidative stress and raises the rate of apoptosis (16, 17). Dysfunction of the lysosomal autophagy pathway can cause damage to various tubular and glomerular cells, thereby accelerating the progression of kidney disease (42).

LOF variants in most genes are recessive. Compensatory mechanisms of organisms often compensate for the effects of heterozygous LOF variants, leading to no marked phenotypic differences between heterozygous LOF and homozygous WT (43). However, a single GOF variant is sufficient to cause disease, making GOF variants primarily inherited in a dominant manner (43). In this study, both the patient and his father carried a heterozygous *ATP6V0A4* p.V512L mutation, with both exhibiting clinical phenotypes of metabolic alkalosis, hypokalemia, and acidic urine, suggesting that the V512L variant is inherited dominantly. Previous research indicated that LOF variants in *ATP6V0A4* cause autosomal recessive dRTA (4), whereas the heterozygous GOF variant identified in our study is inherited as a dominant trait. This may stem from the varied effects of gene variants on protein dosage — functional variants of the same gene can yield distinct and even opposing phenotypes. This phenomenon is called an allelic series (43). For instance, recessive LOF and dominant GOF variants in *FAR1* cause opposing biochemical responses but share similar clinical features, such as developmental delay, spasticity, epilepsy, and cataracts (44). This aligns with the genetic pathogenic mechanisms of LOF and GOF variants in *ATP6V0A4* identified in our study.

Despite sharing the same genotype, the patient and his father exhibited considerable clinical heterogeneity. The observed inconsistency may arise from the fact that numerous deleterious dominant monogenic variants do not conform to classical Mendelian inheritance, allowing individuals with identical genotypes to exhibit markedly different clinical phenotypes or even remain asymptomatic (43). Individuals with a specific genotype (usually heterozygous) who express all phenotypes are termed complete penetrance, and individuals expressing only some phenotypes are termed incomplete penetrance (43, 45). Additionally, some individuals carrying dominant disease-causing alleles may not exhibit the full disease phenotype. Instead, they manifest a “mild phenotype” that lies between the normal and diseased states, a phenomenon known as variable expressivity (43, 45). Notable examples include primary carnitine deficiency (PCD, OMIM 212140) and Fabry disease (Fabry, OMIM 301500). A study published in 2021 compiled a series of cases, including 11 genetic disease cases in which patients and their parents shared the same pathogenic loci, yet the parents did not exhibit the same phenotype (46). Such incomplete penetrance and variable expressivity are prevalent in humans and influenced by various factors, including genetic background, family history, age, sex, and environment (43). This also provides insight into how asymptomatic parents can transmit pathogenic variants to affected offspring.

Currently, no curative or etiological treatments are available for the renal tubular acid-base imbalance caused by *ATP6V0A4* gene mutations (4). Cell and gene therapies for kidney diseases are still in their infancy. Because of the kidney’s diverse specialized cell types and the size-selective filtration function of the glomeruli, efficient drug delivery to the kidneys remains a tremendous challenge, posing a major obstacle to advancing these therapies for kidney diseases (47, 48). With the rapid advancements in artificial intelligence and structural biology, computer-aided drug design demonstrates enormous potential in the development of targeted therapies (49, 50). Building on this, we predicted the structure of the V512L-a4 subunit using AlphaFold and employed structure-based virtual screening to discover F351 (C_25_H_26_FN_3_O_2_S), the inhibitor specifically targeting the V512L-a4 mutant. In vitro studies further verified the effectiveness and safety of F351 in specifically targeting and inhibiting the V512L-a4 mutant. Thus, F351 holds promise as a lead compound for the design and synthesis of highly potent and selective inhibitors targeting the V512L-a4 mutant. This development could pave the way for targeted therapies for patients with renal hydrogen secretion disorders caused by the *ATP6V0A4* p.V512L mutation.

In conclusion, this study established the V512L mutation as a GOF mutation identified in the *ATP6V0A4* gene and successfully employed structure-based virtual screening, experimentally validated, to identify a small-molecule inhibitor that specifically targets the V512L-a4 mutant. The results broaden the phenotypic and mutational spectrum of the *ATP6V0A4* gene and provide valuable insights into potential therapeutic approaches for diseases associated with *ATP6V0A4* mutations.

## Methods

### Sex as a biological variable.

This study exclusively included 2 male patients because of the rarity of the *ATP6V0A4* p.V512L mutation–associated disease, which has thus far only been observed in 2 male individuals.

### Study design.

This study aims to explore the pathogenic mechanisms by which the identified GOF mutation (c.1534G>T, p.V512L) in the *ATP6V0A4* gene contributes to renal dysfunction, hypochloremic metabolic alkalosis, acidic urine, and hypokalemia. Through genetic variation analysis, protein crystallography, and molecular dynamics simulations, we demonstrated that the V512L variant enhanced both protein stability and compactness. We then constructed 2 cell models, WT-a4 and V512L-a4, using HEK293T and M1 cells. HEK293T cells were transfected with plasmids expressing flag-tagged WT-a4 and V512L-a4, and WT-a4 and V512L-a4 M1 cell lines were generated through CRISPR/Cas9 genome editing. In vitro experiments revealed that the V512L variant did not affect *ATP6V0A4* transcription but enhanced protein stability and expression, increasing V-ATPase activity and hydrogen ion transport by renal tubular A-type intercalated cells into the lumen, confirming *ATP6V0A4* p.V512L as a GOF mutation. We further validated that this mutation induced lysosomal dysfunction, leading to blocked autophagic flux, activation of apoptosis, and exacerbation of renal tubular injury. Based on these findings, we combined structure-based virtual screening with in vitro experiments to identify inhibitors targeting the V512L-a4 mutant, providing a foundation for therapeutic exploration of related diseases.

### Patients and clinical examinations.

The patient in this study, diagnosed in December 2021 at the nephrology department of Zhongda Hospital, Southeast University, presented with renal dysfunction, hypochloremic metabolic alkalosis, acidic urine, and hypokalemia, and was found to carry the *ATP6V0A4* p.V512L mutation. The patient’s clinical information was collected, including age, sex, imaging data, blood biochemical tests, urine tests, and kidney pathology results. After clinical evaluation, the patient and his parents underwent whole-exome sequencing and Sanger sequencing for further genetic assessment. The details are provided in the Supplemental Methods.

### Genetic analysis and molecular dynamics simulation.

The ATP6V0A4 protein sequence was downloaded from the National Center for Biotechnology Information database (https://www.ncbi.nlm.nih.gov/) and subjected to conservation analysis using Unipro UGENE v48.1. Potential pathogenicity was predicted using the software tools SIFT, MutPred2, PANTHER, PolyPhen-2, MutationTaster, and CADD. CADD calculated the deleteriousness score of single nucleotide variants (SNVs), with scores higher than 15 considered severely deleterious. We obtained the V-ATPase crystal structure (7UNF) from the PDB (https://www.rcsb.org/structure/7UNF), performed homology modeling of the a4 subunit using SWISS-MODEL (51), and predicted the structure of the V512L-a4 protein with AlphaFold (11). The visualization was performed using the PyMOL 2.5.1 (https://pymol.org/2/). Protein structure quality was assessed using the SAVES web server (https://saves.mbi.ucla.edu/) with tools such as the Ramachandran plot (52), ERRAT (53), and Verify3D (54). Protein stability was predicted using DynaMut software (http://biosig.unimelb.edu.au/dynamut/).

Next, molecular dynamics simulations of the WT and V512L mutant were performed using GROMACS v2023.2. Amber ff14SB (55) force field parameters were applied for the proteins, and the TIP3P model was used for water. Each system was solvated in a dodecahedral water box using the TIP3P water model, and the appropriate number of sodium or chloride ions was added to neutralize the system. Periodic boundary conditions were applied in all 3 dimensions. First, energy minimization was conducted using the steepest descent method, followed by pre-equilibration of the system using constant-temperature, constant-volume (NVT) and constant-temperature, constant-pressure (NPT) ensembles. NVT equilibration was performed at a stable temperature of 300 K, utilizing a velocity-rescale thermostat with a time constant of 0.1 ps for 100 ps. Next, the Berendsen barostat was used to maintain a pressure of 1 bar, with a time constant of 2.0 ps for 100 ps of NPT equilibration. Electrostatic interactions were managed using the SPME algorithm with a 12 Å cutoff for real-space interactions. The LINCS algorithm was applied to constrain all bonds involving hydrogen atoms. Molecular dynamics simulations were conducted with a 2 fs time step, recording conformations every 10 ps, with each system simulated for 100 ns. The trajectories were analyzed for RMSD, Rg, RMSF, and the free energy landscape. Changes in protein secondary structure during the molecular dynamics simulations were assessed using DSSP software.

### Plasmid construction and transduction.

The coding sequences of the human *ATP6V0A4* gene (NM_020632.3), including both the WT and the c.1534G>T variants, were synthesized by GenScript Biotech Corporation. The synthesized gene fragments were digested with KpnI and cloned into the pcDNA3.1(+)-N-eGFP vector using homologous recombination. Recombinant plasmids were plated on ampicillin agar for positive clone screening. Sequencing verified single clones, and correct plasmids were confirmed by MluI and SmaI double digestion to ensure proper size. Supplemental Figure 10 lists the plasmids used in this study, and Supplemental Table 10 lists the primer sequences for plasmid construction.

HEK293T cells were obtained from ATCC and cultured in DMEM (Gibco) with 10% FBS at 37°C and 5% CO_2_. Plasmids were transiently transfected into HEK293T cells using Lipofectamine 3000 (Invitrogen). Eight hours after transfection, the medium was replaced with fresh DMEM. Transfection efficiency was assessed by epifluorescence microscopy (Olympus IX73) 24 hours after transfection, and cells were harvested for subsequent experiments 48 hours after transfection.

### Generation of stable V512L-transduced M1 cell lines.

M1s were purchased from ATCC. The WT and V512L-transduced M1s were produced using CRISPR/Cas9 technology. First, sgRNA sequences targeting mouse *ATP6V0A4* were designed using CRISPR DESIGN (https://portals.broadinstitute.org/gppx/crispick/public). After Cas9 induced DNA double-strand breaks, human *ATP6V0A4* gene sequences, including both WT and the pathogenic c.1534G>T mutant, were provided as repair templates to facilitate knockin of the gene into M1 cells. Using the pU6-(BbsI)-CBh-Cas9-T2A-BFP (64323) empty plasmid as a backbone, a targeting plasmid expressing the sgRNA was constructed. A repair template plasmid was also generated by recombining 1 kb sequences flanking the target site with the synthesized *ATP6V0A4*-P2A-puromycin sequence. After sequencing, the constructs were cotransfected into M1 cells. Positive clones were selected with puromycin and expanded after DNA verification. Finally, M1 cells stably expressing WT and V512L-a4 were cultured in 10% FBS, 1% P/S, 1 μg/mL puromycin, and DMEM/F12, at 37°C and 5% CO_2_.

### Western blot.

Total proteins were extracted by incubating cells in RIPA lysis buffer (BioSharp), and protein concentrations were determined with a BCA protein assay kit (KeyGEN BioTECH). Protein extracts were separated by gel electrophoresis, transferred to PVDF membrane, and incubated with blocking solution (NCM Biotech) for 30 minutes. The membrane was incubated with primary antibodies overnight at 4°C, washed in TBST, incubated with secondary antibodies at room temperature for 1 hour, and washed with TBST, then developed with ECL (BioSharp). The following primary antibodies were used for Western blot: ATP6V0A4 (orb666101, Biorybt), β-actin (sc-47778, Santa Cruz Biotechnology), P62 (18420-1-AP, Proteintech), LC3 (14600-1-AP, Proteintech), Bax (89477S, Cell Signaling Technology), cleaved caspase-3 (9661S, Cell Signaling Technology), and Bcl-2 (3498S, CST).

### Protein stability assay.

For CHX chase experiments, M1 cells were treated with 100 μg/mL CHX (HY-12320, MedChemExpress) and were collected at each indicated time point (0, 2, 4, 8, 12, 24 hours) for immunoblot analysis.

### V-ATPase and H^+^-K^+^-ATPase activity measurement.

V-ATPase and H^+^-K^+^-ATPase activity was assayed using the V-ATPase activity assay kit (GMS50247.1, GENMED) and H^+^-K^+^-ATPase activity assay kit (GMS50243.1.1, GENMED). Total protein was extracted from collected cells for quantification. GENMED buffer, enzyme solution, reaction solution, and substrate solution were sequentially added to a microplate. The mixture was incubated at 37°C for 3 minutes; then, 100 μg of protein was added, mixed, and immediately placed in a microplate reader to measure absorbance at 340 nm every 2 minutes for 20 minutes.

### Virtual screening of V512L-a4 mutant inhibitors.

A structure-based virtual screening approach was adopted to discover *ATP6V0A4* p.V512L inhibitors. First, the ligand and receptor files were prepared. We employed the ATP6V0A4 p.V512L mutant protein structure as the receptor for docking, with preprocessing conducted using AutoDock Tools (21), which involved adding hydrogen atoms, assigning charges, and converting formats. We then utilized the CavityPlus (18) website along with the mutated residue position to predict the binding pocket of the mutant. The ligand library comprises over 2 million compound molecules sourced from the ChemDiv chemical database, the COCONUT natural product database (19), the MCE bioactive compound database, and the DrugBank database (20). Ligand structures were downloaded in SDF format from official websites, with important chiralities preserved. Compounds were filtered for PAINS and alerts (56). Gypsum-DL (57) was used to generate ionization states of the compounds at pH 7.0 ± 2.0 and produce one low-energy conformation for each ligand, and Meeko was employed to convert the compound structures into the pdbqt format.

Virtual screening of the preprocessed compounds was conducted using AutoDock Vina 1.2.5. Lower docking scores reflect stronger binding affinity between the receptor and ligand. The top 5,000 scoring molecules were selected for clustering (22), yielding 100 cluster center compounds. Each of these 100 compounds was subjected to a 10 ns molecular dynamics simulation, using the docking pose as the initial structure for the simulation. In the molecular dynamics simulations, RESP2 (0.5) charges for the small molecules were generated using Multiwfn (58), and Sobtop (http://sobereva.com/soft/Sobtop/) was employed to generate the topology and gro files for GROMACS (59), with the GAFF force field applied (60). All other molecular dynamics parameters and procedures were consistent with those previously described (see Genetic analysis and molecular dynamic simulation). Binding free energies were calculated from the final 1 ns of the simulation trajectory.

Finally, we used the molecular mechanics/generalized born surface area (MM/GBSA) method to calculate the free energy of the protein-ligand complexes. Binding free energy (ΔG_bind_) is broken down into various energy components, as follows (61):

ΔG_bind_ = ΔG_bind,gas_ + (ΔG_COM,sol_ − ΔG_REC,sol_ − ΔG_LIG,sol_) = ΔG_bind,gas_ + ΔG_solvation_

ΔG_bind,gas_ = ΔH − TΔS ≈ ΔE_MM_ − TΔS

ΔG_bind_ ≈ ΔE_MM_ + ΔG_sol_ − TΔS

ΔE_MM_ = ΔE_int_ + ΔE_vdw_ + ΔE_ele_

ΔG_sol_ = ΔG_GB_ + ΔG_SA_

ΔG_SA_ = γ ∙ SASA + b

ΔG_bind_ is approximated as the sum of molecular mechanics energy (ΔE_MM_), solvation energy (ΔG_sol_), and conformational entropy − TΔS. ΔE_MM_ comprises internal energy (ΔE_int_), van der Waals energy (ΔE_vdw_), and electrostatic energy (ΔE_ele_). ΔG_sol_ sums polar solvation term ΔG_GB_ and nonpolar solvation term ΔG_SA_. ΔG_GB_ is estimated by the Generalized Born model, and ΔG_SA_ is calculated using solvent-accessible surface area (SASA). MM/GBSA calculations were conducted with the gmx MMPBS tool (23, 24).

### Functional validation of V512L-a4 mutant inhibitors.

Selected compounds were supplied by in-house synthesis at TargetMol or ChemDiv: relamorelin (T34281, TargetMol), forsythiaside A (T3670, TargetMol), PAR-1AC (T38836L, TargetMol), F359-0497 (ChemDiv), and F351-0364 (ChemDiv). M1 cells and HEK293T cells were treated with indicated concentrations of selected small-molecule compounds and collected at each indicated time point for V-ATPase activity, H^+^-K^+^-ATPase activity evaluation, immunoblot analysis, lysosomal pH, and lysosomal function assessment. The cytotoxicity of the tested compounds on M1 cells and HEK293T cells was determined by the CCK8 (C0005, TargetMol).

### Other methods.

The detailed experimental procedures for whole-exome sequencing, Sanger sequencing, IHC, immunofluorescence, Western blot, RT-qPCR, lysosomal pH, endosomal pH, lysosomal function assessment, autophagic flux assessment, ROS measurement, mitochondrial membrane potential assessment, transmission electron microscopy, and the CCK8 assay are provided in the Supplemental Methods.

### Statistics.

All data are presented as mean ± SD. The number of replicates for each experiment is shown in the figure legends. Statistical analysis was performed using GraphPad Prism 9.0 software. Differences between groups were compared with a 2-tailed, unpaired *t* test (2 groups) when datasets were normally distributed and Mann-Whitney *U* test when they were not normally distributed. For the comparison of 3 groups, 1-way ANOVA was used. All *P* values of less than 0.001 were considered statistically significant, as indicated in the text.

### Study approval.

This study was approved by the Ethics Committee of Affiliated Zhongda Hospital of Southeast University (approval 2024ZDSYLL351-P01) and conducted in strict accordance with the Declaration of Helsinki. Written informed consent was obtained from the patients or their families.

### Data availability.

All data needed to evaluate the conclusions in the paper are present in the paper and/or the supplement. The raw next-generation sequencing data reported here have been deposited in the Genome Sequence Archive in National Genomics Data Center, China National Center for Bioinformation/Beijing Institute of Genomics, Chinese Academy of Sciences (GSA-Human: HRA011176; publicly accessible at https://ngdc.cncb.ac.cn/gsa-human). Values for all data points in graphs are reported in the Supporting Data Values file.

## Author contributions

SQP and BW conceived and designed the study. SQP performed in vitro experiments, analyzed data, and drafted the manuscript. QQW assisted with in vitro experiments and revision of the article. WYW and CKW constructed the stable WT and V512L-transduced M1 cells. RNZ, JL, and LH assisted in virtual screening of targeted V512L-a4 inhibitors. YLZ, JXW, WS, JLY, XXW, ZYW, HF, HC, JXS, and YY provided technical assistance and material support. XHL and TSL provided valuable help and support in the search for additional families. BCL provided experimental sites and equipment and provided financial support. XLZ supervised the study and provided financial support. BW designed the experiments, directed and supervised the study, and provided financial support.

## Supplementary Material

Supplemental data

Unedited blot and gel images

Supporting data values

## Figures and Tables

**Table 1:**
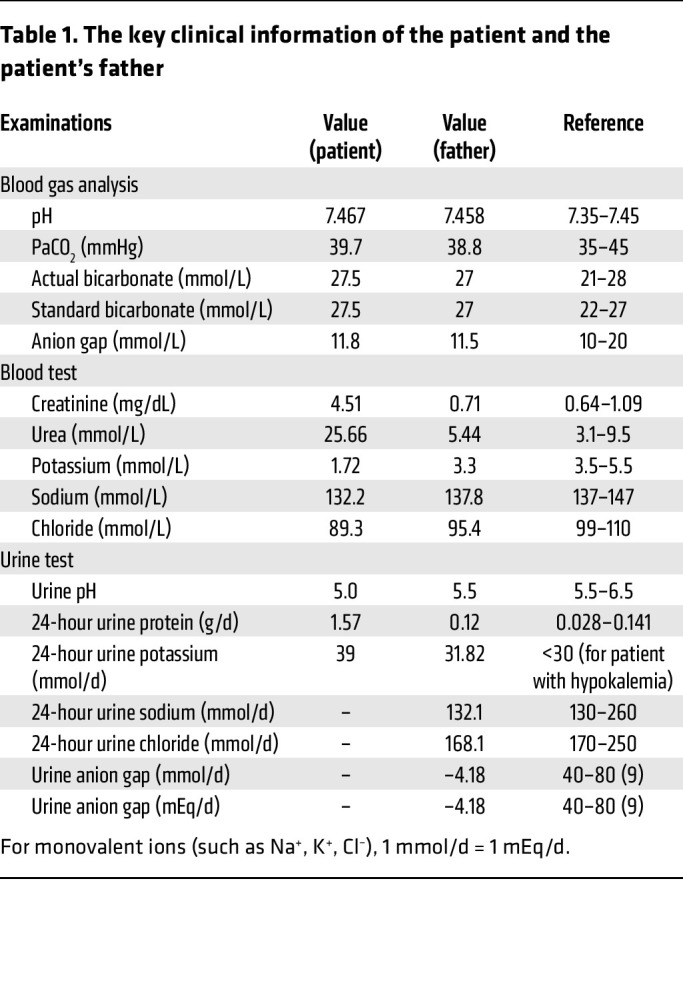
The key clinical information of the patient and the patient’s father

## Version 1 04/29/2025

In-Press Preview

## Version 2 07/01/2025

Electronic publication. Includes correct version of 2G.
