# Supplementary material for: A gain-of-function mutation in ATP6V0A4 drives primary distal renal tubular alkalosis with enhanced V-ATPase activity
Source: J Clin Invest. 2025 Apr 29;135(13):e188807. doi: 10.1172/JCI188807 (PMC12208546; doi:10.1172/JCI188807)
Supplement: Supplemental data [file jci-135-188807-s244.pdf]

Supplementary Materials for

A gain-of-function mutation in ATP6V0A4 drives primary distal renal tubular alkalosis with enhanced V-ATPase activity

Si-qi Peng[1†], Qian-qian Wu[1†], Wan-yi Wang[2], Yi-Lin Zhang[1], Rui-ning Zhou[3], Jun Liao[4], Jin-xuan Wei[1], Yan Yang[1], Wen Shi[1], Jun-lan Yang[1], Xiao-xu Wang[1], Zhi-yuan Wei[1], Jia-xuan Sun[1], Lu Huang[3], Hong Fan[5], Hui Cai[6], Cheng-kun Wang[2], Xin-hua Li[7], Ting-song Li[8], Bi-cheng Liu[1*], Xiao-liang Zhang[1]* and Bin Wang[1]*

[1]Department of Nephrology, Zhong Da Hospital, Southeast University School of Medicine, Nanjing, 210009, Jiangsu, China.

[2]Department of Physiology, School of Basic Medical Science, Nanjing Medical University, Nanjing, 211166, Jiangsu, China.

[3]Nanjing Jiangbei New Area Biopharmaceutical Public Service Platform Co. Ltd, Nanjing, 210000, Jiangsu, China.

[4]School of Science, China Pharmaceutical University, #639 Longmian Avenue, Jiangning District, Nanjing, 211198, Jiangsu, China.

[5]Department of Medical Genetics and Developmental Biology, Medical School of Southeast University, The Key Laboratory of Developmental Genes and Human Diseases, Ministry of Education, Southeast University, Nanjing, 210009, China.

[6]Department of Medicine, Renal Division, Emory University School of Medicine, Atlanta, GA, USA.

[7]Department of Infectious Diseases, Third Affiliated Hospital of Sun Yat-sen University, Guangzhou, 510630, Guangdong, China.

[8]Department of neurology, Children's Hospital of Chongqing Medical University, Chong Qin, 400000, China.

†These authors contributed equally to this work.

Conflict of interest: The authors have declared that no conflict of interest exists.

*Correspondence to:
Bin Wang, Department of Nephrology, Zhong Da Hospital, Southeast University School of Medicine, 87 Ding Jia Qiao Road, Nanjing 210009, Jiangsu, China. Email: wangbinhewei@126.com, telephone number: +8613372253855;
Xiao-liang Zhang, Department of Nephrology, Zhong Da Hospital, Southeast University School of Medicine, 87 Ding Jia Qiao Road, Nanjing 210009, Jiangsu, China. Email: tonyxlz@163.com, telephone number: +8613852290358;
Bi-cheng Liu, Department of Nephrology, Zhong Da Hospital, Southeast University School of Medicine, 87 Ding Jia Qiao Road, Nanjing 210009, Jiangsu, China. Email: liubc64@163.com, telephone number: +8618001580838;

The PDF file includes:

Material and Methods
Supplementary Figure S1-S10
Supplementary Table S1-S10

Materials and methods

1.WES and Sanger sequencing

Sequencing was done by MyGenostics, China. DNA was extracted from the peripheral venous blood samples (3 ml) from the patient and his partents.Genomic DNA was extracted using the DNeasy Blood & Tissue Kit (Qiagen, Germany) according to the manufacturer's protocol. A targeted sequence capture chip (MyGenostics, GenCap) was employed to capture all exons from 23,000 genes. Whole exome sequencing was then conducted on a DNBSEQ-T7 sequencer (BGI, China). Candidate variants identified from the sequencing data were validated through family-based verification using Sanger sequencing. The sequencing results were subsequently analyzed with DNASTAR (Madison) software.The primer sequences used are as follows: forward primer: TATGGAAAATGGGGCTGGGT, reverse primer:GCCCCGGTTGATCATTGTTT.

2. Immunohistochemistry

Paraffin-embedded kidney tissue sections (4 μm) were deparaffinized, dehydrated, and subjected to antigen retrieval using EDTA. The sections were then incubated with 1% hydrogen peroxide for 30 minutes, blocked with 10% goat serum for 30 minutes, and incubated with the primary antibody overnight at 4℃. Antigen-antibody binding was detected using an immunohistochemistry kit (Maixin), according to the manufacturer’s instructions, and sections were visualized using an Nikon microscope. Antibodies specific for IHC: ATP6V0A4 (ab204737, Abcam), P62 (ab207305, Abcam).

3. Immunofluorescence

Paraffin-embedded kidney tissue sections (4um) were routinely dewaxed, dehydrated, and antigen retrieval was performed using EDTA solution. After blocking with 10% goat serum for 30 minutes at room temperature, primary antibodies were applied and incubated overnight at 4° C. Fluorescently labeled secondary antibodies were then added and incubated for 1 hour at room temperature in the dark. Nuclei were stained with DAPI for 10 minutes, and the sections were mounted and imaged using a laser confocal fluorescence microscope (Olympus).

Cells on coverslips were fixed with 5% paraformaldehyde for 10 minutes, permeabilized with 0.25% Triton X-100 for 5 minutes, and blocked with 10% goat serum for 20 minutes. Primary antibodies were added and incubated overnight at 4°C. The next day, fluorescently labeled secondary antibodies were incubated at room temperature for 1 hour in the dark. After three PBS washes, cells were stained with DAPI for 10 minutes, mounted, and imaged using a confocal fluorescence microscope (Olympus). Primary antibodies used for IF included:

ATP6V0A4 (ab204737, Abcam), ATP6V0A4 (21570-1-AP, Proteintech), LAMP2 (66301-1-Ig, Proteintech), LC3 (14600-1-AP, Proteintech).

4. Real-time quantitative polymerase chain reaction(RT-qPCR)

Total RNA was extracted using the Trizol reagent kit (Invitrogen, USA). The RNA was subsequently reverse-transcribed into cDNA using the RNA reverse transcription kit (Novozan， China). The relative mRNA expression was then measured using a real-time fluorescent PCR system (Novozan, China). The FastStart Universal SYBR Green Master was added to each polymerase chain reaction along with cDNA and specific primers to a total volume of 10 ul. RT-qPCR was performed on7300 Real-Time PCR system (Applied Biosystems)。

5. Lysosomal pH

Wild-type and V512L M1 cells were seeded in confocal dishes. After adhesion, Bafilomycin A1 (HY-100558, MCE) was diluted to 50 nmol/L and added to the V512 cell culture medium as a control, followed by incubation at 37°C for 4 hours. LysoTracker Red DND-99 (L12492, Invitrogen) was diluted to 100 nmol/L and LysoSensor Yellow/Blue DND-160 (L7545, Invitrogen) to 2 μmol/L using pre-warmed culture medium. The solutions were then incubated with cells at 37°C for 30 minutes. After incubation, the medium was replaced with fresh medium. LysoTracker fluorescence was measured at 577/590 nm and LysoSensor fluorescence at 340/440 nm and 380/535 nm using a laser confocal microscopy (Olympus). The pH value was quantified using an intracellular pH calibration buffer kit (Invitrogen).

Additionally,We used pHLys Red (L265, Dojindo) and LysoPrime Green (L261, Dojindo) kits to monitor lysosomal pH. pHLys Red fluorescence decreases as lysosomal function declines (pH shifts from acidic to neutral), while LysoPrime Green fluorescence remains stable. After cell adhesion, diluted LysoPrime Green and pHLys Red were added sequentially and incubated at 37°C for 30 minutes. Fluorescence intensities were measured using a laser confocal microscopy (Olympus). at 562/586 nm for pHLys Red and 456/540 nm for LysoPrime Green.

6. Endosomal pH

The ECGreen kit (E296, Dojindo) and PlasMem Bright Red (P505, Dojindo) were used to detect endosomal pH. ECGreen is a cell membrane-impermeable fluorescent dye that remains on the membrane. Its fluorescence increases in the acidic environment of endosomes after endocytosis. After cell adhesion, ECGreen and PlasMem Bright Red were diluted, added, and incubated with cells at 37°C for 30 minutes. The fluorescence intensities were measured using a laser confocal microscopy (Olympus) at 386/522 nm for ECGreen and 561/560-700 nm for PlasMem Bright

Red.

7. Lysosomal hydrolase activity

Lysosomal hydrolase activity was measured using DQ Red-BSA (D12051, Invitrogen). Wild-type and V512L M1 cells were seeded in confocal dishes. After adhesion, Bafilomycin A1 was diluted to 50 nmol/L and added to the mutant cell culture medium as a control. The cells were incubated with 10 μg/ml DQ-BSA in the dark at 37°C for 12 hours. After incubation, DQ-BSA was removed, and the cells were washed twice with pre-warmed PBS. Nuclear staining reagent (H342, Dojindo) was then added, and the cells were incubated in the dark at 37°C for 15 minutes, followed by two washes with pre-warmed PBS. Fluorescence images were captured using a laser confocal microscopy(Olympus) at 561 nm/560-650 nm for DQ Red-BSA and 350 nm/460 nm for nuclear staining.

Lysosomal hydrolase activities of cathepsin D and cathepsin B were measured according to the instructions provided in the Cathepsin D Activity Assay Kit (ab65302, Abcam) and Cathepsin B Activity Assay Kit (ab65300, Abcam).

8. Autophagic flux assay

Autophagic flux was evaluated by detecting autophagosomes using the DAPRed kit (D677, Dojindo) and assessing autolysosome formation using the DALGreen kit (D675, Dojindo), following the manufacturer's instructions.Wild-type and V512L M1 cells were seeded at an appropriate density in confocal dishes. After cell adhesion, Bafilomycin A1 was diluted to 50 nmol/L and added to the V512L cell culture medium as a control, followed by incubation at 37° C for 8 hours. The cells were then washed three times with pre-warmed PBS. Subsequently, DAPRed (0.2 μmol/L) and DALGreen (1 μmol/L) working solutions were added, and the cells were incubated in the dark at 37°C for 30 minutes. After incubation, the medium was discarded, and the cells were washed twice with pre-warmed PBS. Fluorescence images were captured using a laser confocal microscope (Olympus) at 530 nm/720 nm for DAPRed staining and at 405 nm/525 nm for DALGreen staining.

10.Transmission electron microscopy

For studies involving electron microscopy (EM), cells are collected after centrifuge and the precipitation, then fixed using an EM fixation buffer containing 2.5% glutaraldehyde in 0.1 M phosphate buffer at pH 7.4, followed by treatment with 1% osmic acid for 2 h . Subsequently, the cells underwent dehydration, sectioning into thin slices. Ultrathin sections (60-80 nm) were cut, collected on 150 mesh copper grids, and stained with 2% uranium acetate saturated alcohol

solution and 2.6% lead citrate.Image acquisition and analysis were performed using a transmission electron microscope (HT7800, Hitachi).

9. Intracellular ROS detection

Generation of intracellular ROS was detected using a ROS-sensitive fluorescent probe (DCFH-DA). After cell adhesion, the cells were incubated with working solutions of Highly Sensitive DCFH-DA Dye (R252, Dojindo) and PlasMem Bright Red (P505, Dojindo) at 37° C in the dark for 30 minutes. Following incubation, the medium was discarded, and the cells were washed twice with pre-warmed PBS. Fluorescence images were captured using a laser confocal microscope (Olympus) at 488 nm/500-550 nm.

12. Mitochondrial membrane potential

JC-1 is a widely used small-molecule probe for mitochondrial membrane potential, accumulating in mitochondria in a membrane potential-dependent manner. As JC-1 aggregates, its fluorescence shifts from green (530 nm) to red (590 nm). During mitochondrial depolarization, the red-to-green fluorescence intensity ratio decreases. Mitochondrial membrane potential was measured using the JC-1 MitoMP detection kit (MT09, Dojindo). After cell adhesion, cells were washed three times with pre-warmed PBS, followed by incubation with JC-1 working solution and nuclear staining reagent (H342, Dojindo) in the dark at 37°C for 1 hour. The medium was then discarded, and cells were washed twice with pre-warmed PBS Fluorescence images were captured using a laser confocal microscope (Olympus) at 488 nm/500-550 nm and 561 nm/560-610 nm after adding Imaging Buffer Solution.

13. Cell-counting Kit 8 (CCK-8) cell viability assay

CCK8 (C0005,TargetMol) assays were used to detect the cytotoxicity of compounds.WT and V512L M1 cells (100 μl) were seeded in 96-well plates and incubated at 37°C until density reached 60%-70%.The supernatant was removed, and CCK-8 was added at 1/10 volume.The mixture was thoroughly mixed, then incubated for 4 hours.Absorbance at 450 nm was measured at 0.5, 1, 2, and 4 hours to assess cell viability.

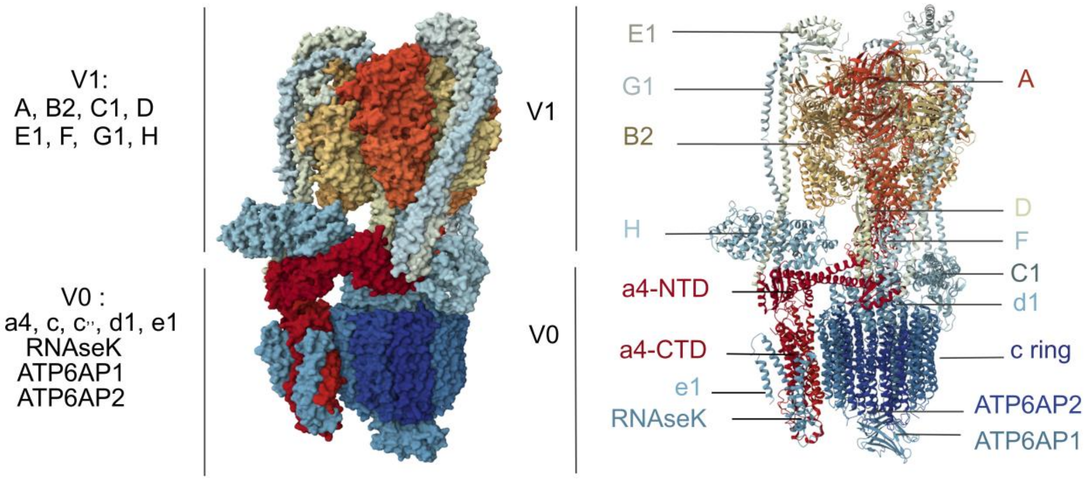

Figure S1. Schematic diagram of the ultrastructure of human V-ATPase. Note: Sourced from the PDB database https://www.rcsb.org/, PDB ID 7UNF.

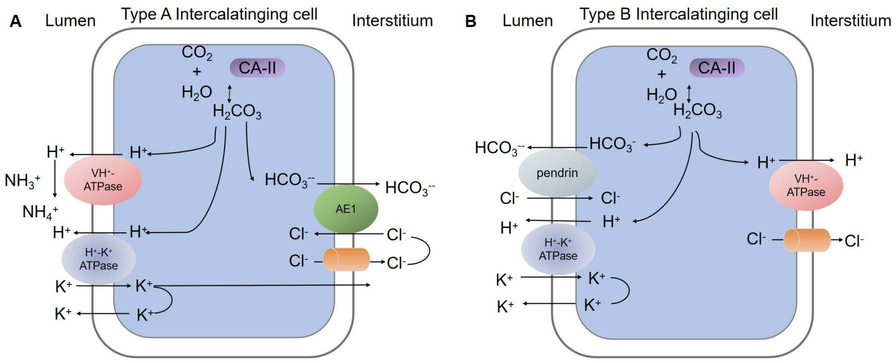

Figure S2. Acid-base transporters in type A intercalated cells **(A)** and type B intercalated cells **(B)** in the distal renal tubule and collecting duct. Note: CA-II, carbonic anhydrase II. VH^+-ATPase, V-ATPase. AE1, $Cl^-/HCO3^-$ transporter encoded by the *SLC4A1* gene. pendrin, $Cl^-/HCO3^-$ exchanger encoded by the *SLC26A4* gene.

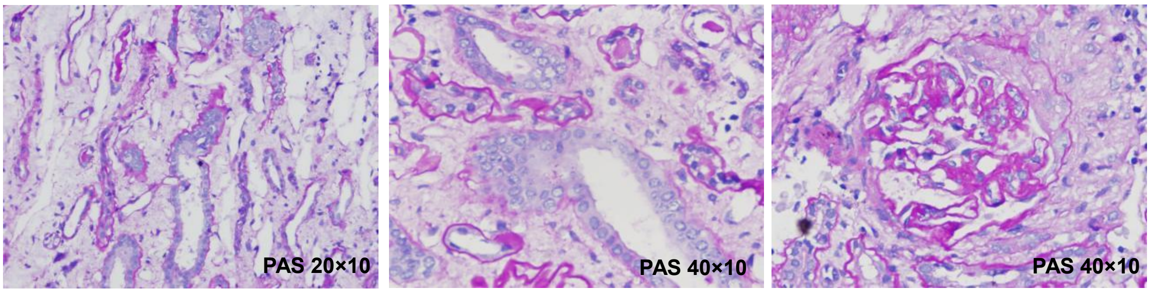

Figure S3. The pathology views of the renal biopsy specimens. Pathological characteristics: tubular atrophy/interstitial fibrosis (85%)，global glomerulosclerosis (6/21),

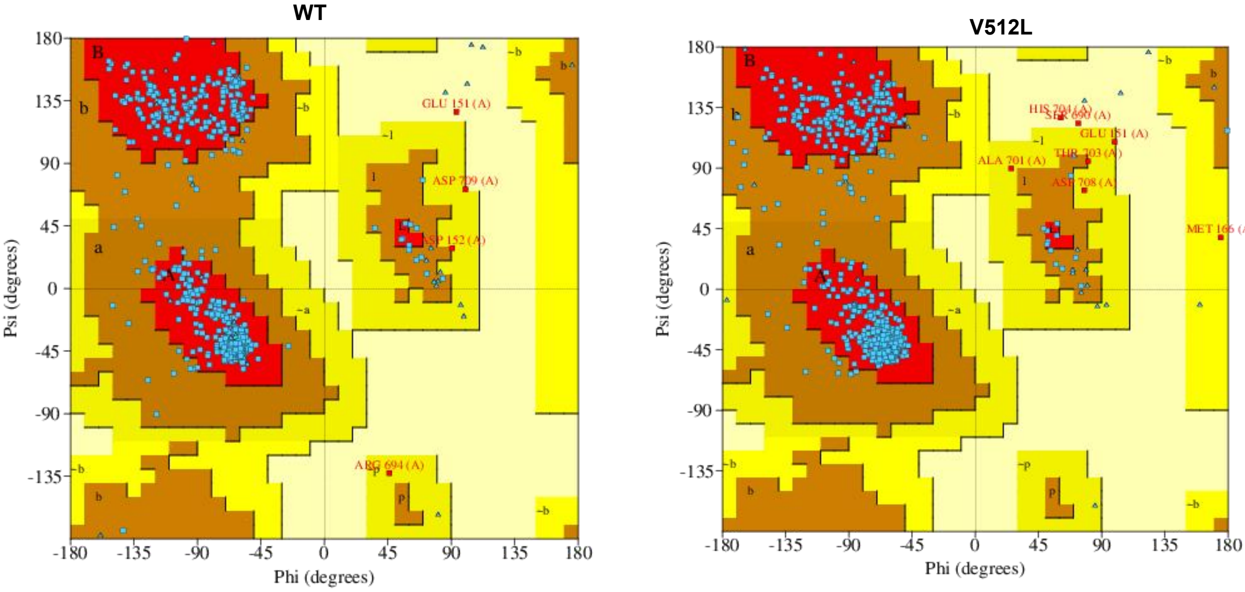

Figure S4. Ramachandran plots of the WT-a4 and V512L-a4 proteins. The Ramachandran plot analysis conducted via the Procheck web server shows that more than 90% of the amino acid residues lie within the core and generally allowed regions, indicating that both WT-a4 and V512L-a4 subunits structures are of high quality. **Note:** The x and y axes represent phi and psi, corresponding to the two dihedral angles (phi, C-N-Cα-C) and (psi, N-Cα-C-N) of the amino acid unit. The **red** region indicates the core conformational zone, the **brown** zone represents the allowed zone, the **yellow** zone represents the marginally allowed conformational zone, and the **white** zone indicates the disallowed angle area. The **blue** dots denote the Phi and Psi angles of each residue's (amino acid) main chain in the protein or polypeptide. If most dots fall in the red and brown areas, it suggests a reasonable protein structure.

A

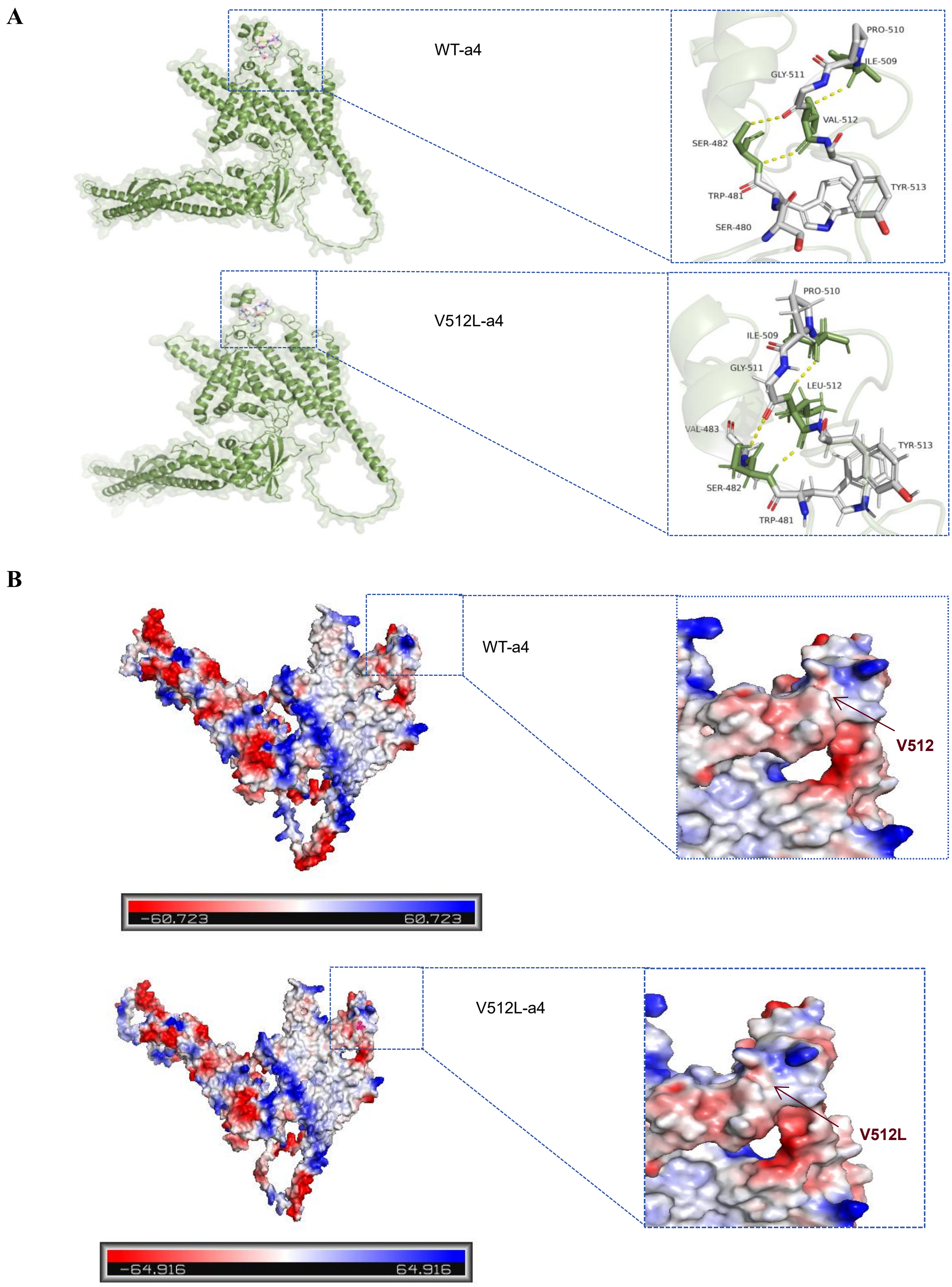

Figure S5. Structural analyses of WT-a4 and V512L-a4 proteins. **(A)** Compared to the WT-a4, the hydrogen bond distance between the VAL-512 and TRP-481, ILE-509 amino acid residues in the

V512L-a4 is reduced, resulting in a stronger interaction. **(B)** Using PyMOL software (Version 4.6), we mapped the predicted electrostatic potential of the protein onto its surface, coloring it in a gradient from red (negative) to blue (positive). The results showed no change in the surface potential between the V512L-a4 and the WT-a4 both exhibiting a neutral potential distribution, indicating that the mutant can maintain a highly stable conformation, thereby enhancing its overall stability.

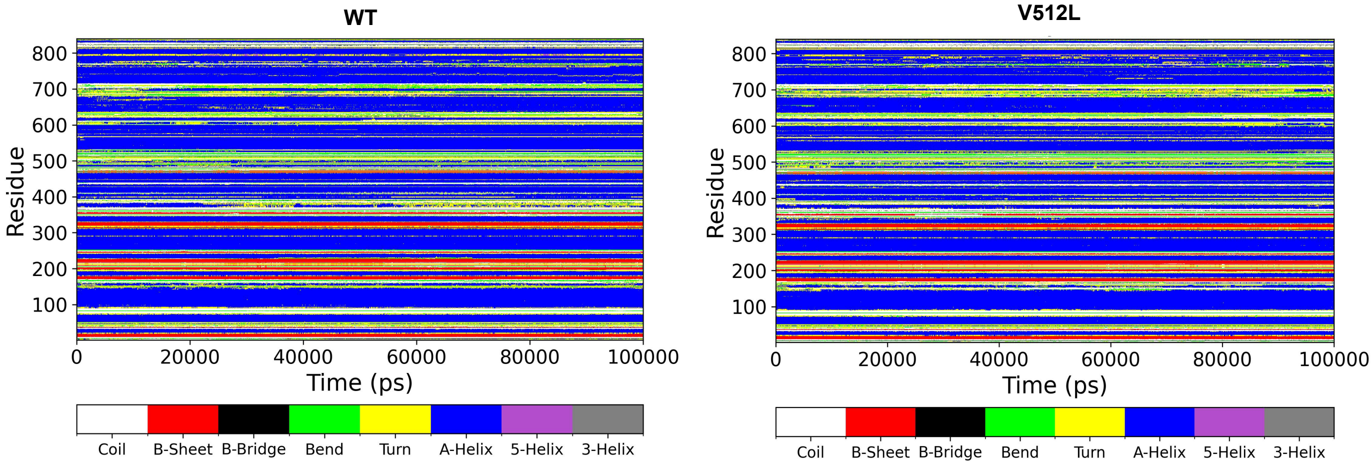

Figure S6. Secondary structure changes in WT-a4 and V512L-a4 subunits. The changes in the secondary structure of the protein during the 100 ns molecular dynamics(MD) simulation were calculated using the DSSP software. Compared to the WT-a4 protein, the secondary structure of the V512L-a4 protein did not exhibit significant changes.

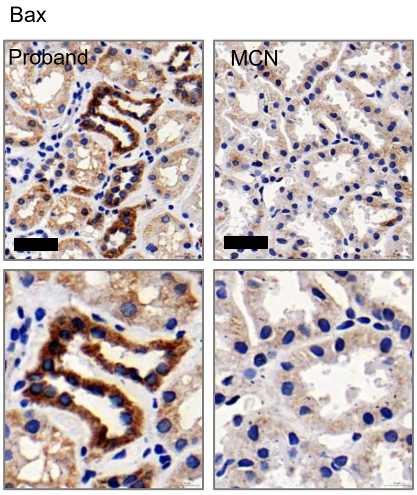

Figure S7. Immunohistochemistry confirmed patient renal tubular tissues with increased bax expression abundance compared with MCN. Scale bar, 40um.

。

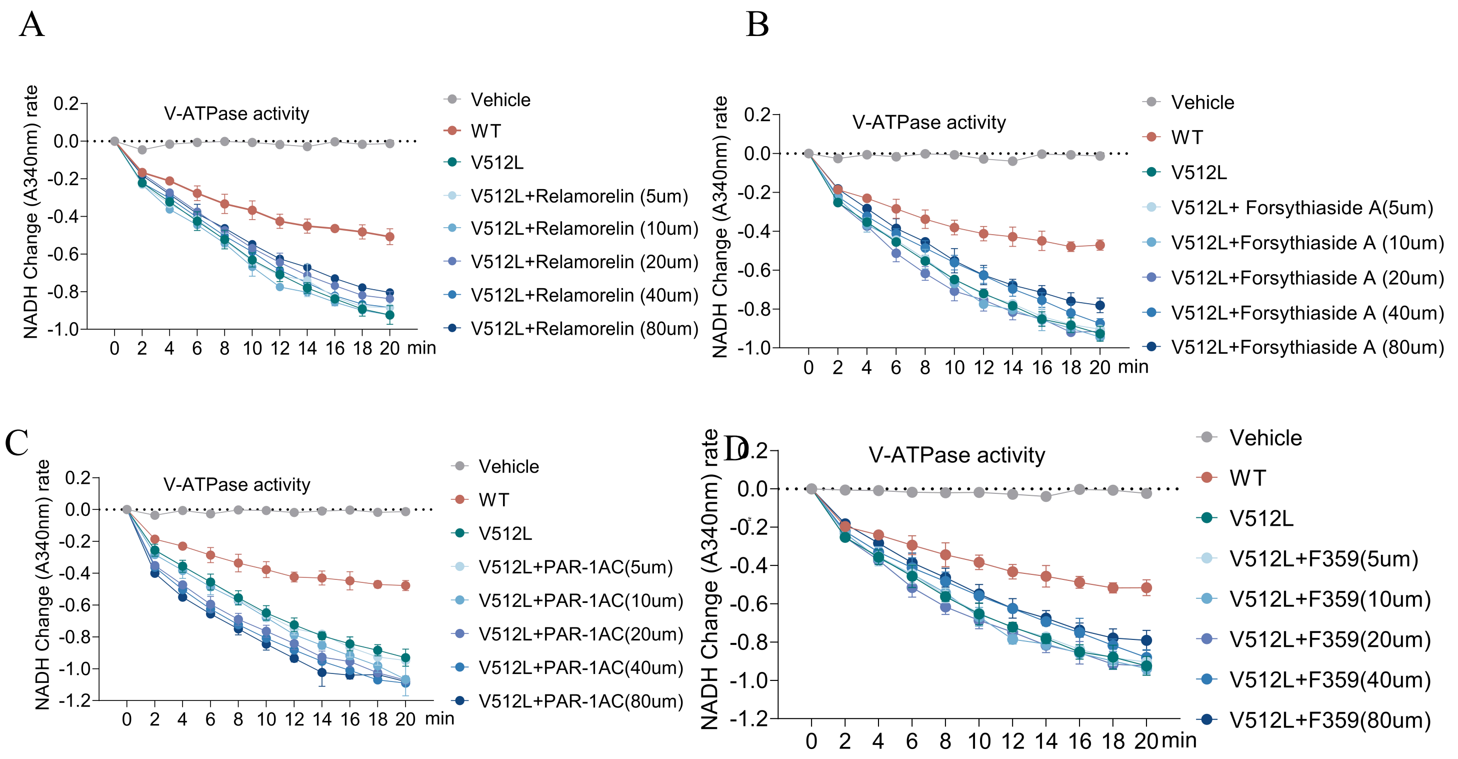

Figure S8. The effects of different compounds on V-ATPase activity in V512L-transduced M1s. **(A)** The V-ATPase activity analysis of M1 cells treated with vehicle or relamorelin (C43H50N8O5S). **(B)** The V-ATPase activity analysis of M1 cells treated with vehicle or forsythiaside A (C29H36O15). **(C)** The V-ATPase activity analysis of M1 cells treated with vehicle or protease-activated receptor-1, PAR-1 Agonist acetate (PAR-1AC) (C35H58N10O9.C2H4O2). **(D)** The V-ATPase activity analysis of M1 cells treated with vehicle or F359 (ChemDiv ID: F359-0497, MolFormula:C26H22FN5O3S). Data are given as mean ± SEM, n = 3.

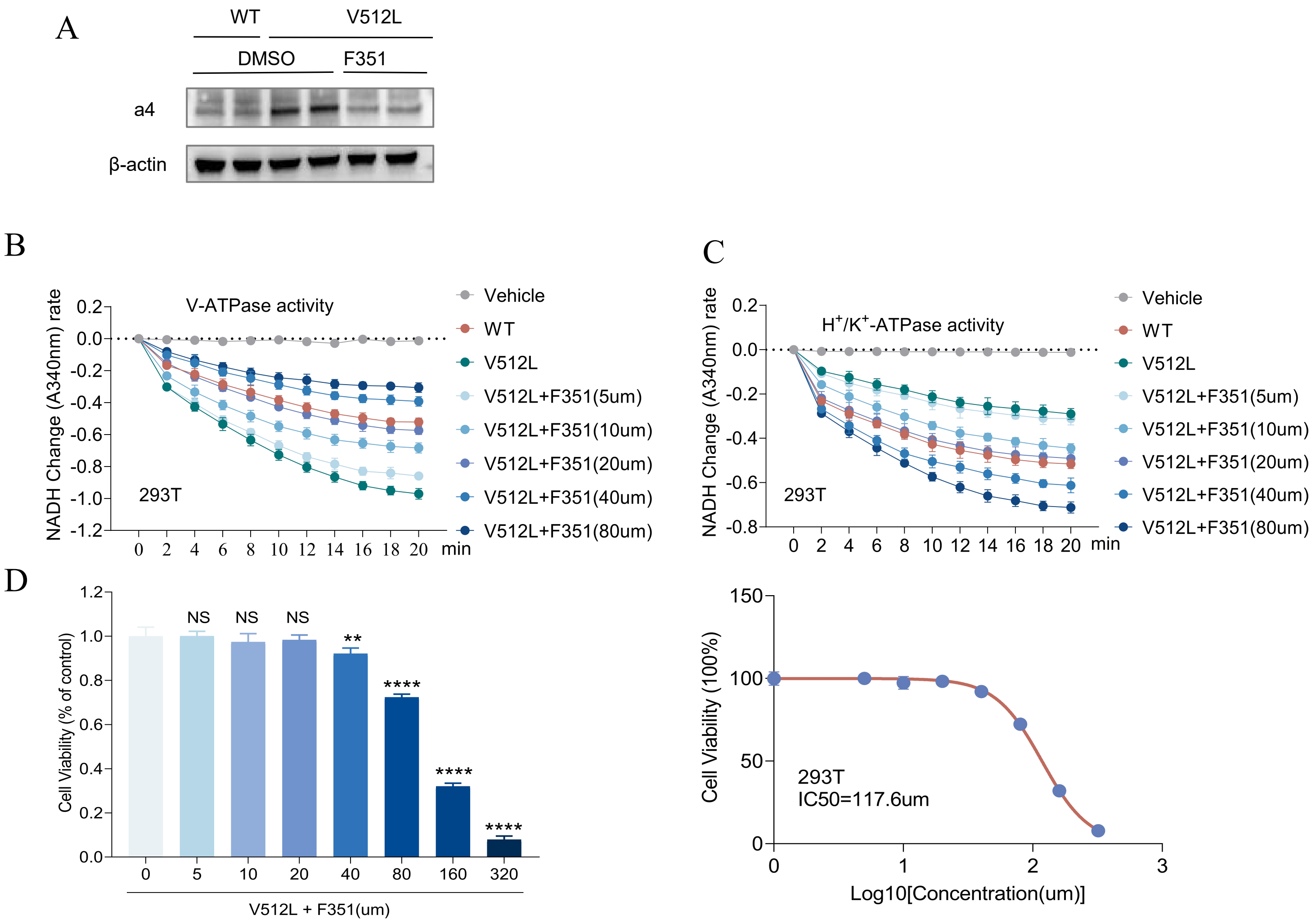

Fig S9. The effects of F351(ChemDiv ID: F351-0364, MolFormula:C25H26FN3O2S) on V-ATPase activity, H^+-K^+-ATPase activity and cell viability in 293T cells transfected with V512L-a4 expression plasmids. (A) The ATP6V0A4 expression levels analysis of 293T cells treated with DMSO or F351 for 24h. DMSO was added as solvent control. (B) The V-ATPase activity analysis of 293T cells treated with vehicle or F351 for 24h. n = 3. (C) The H^+/K^+-ATPase activity analysis of 293T cells treated with vehicle or F351 for 24h. n=3. (D) The cell viability of 293T cells treated with vehicle or F351 of indicated concentration for 24h, Measured by CCK8 assay (n=6 per group). 293T, human embryonic kidney cell 293T. Each group was always compared to the first group, which was considered as the reference group. All data are given as mean ± SEM. **P＜0.005, ****P < 0.0001.

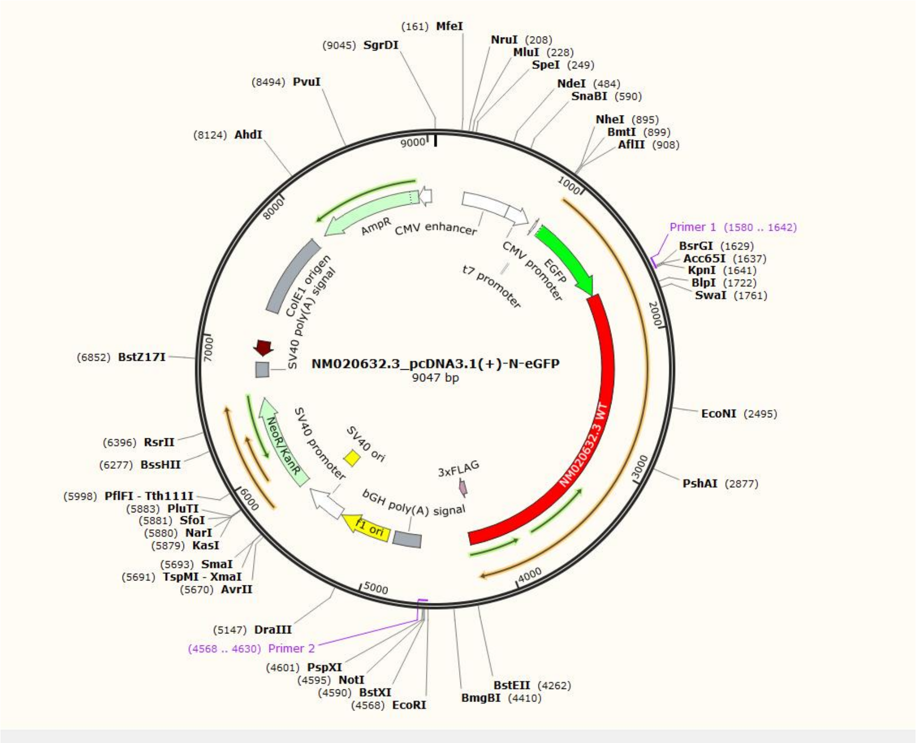

Figure S10. The pcDNA+N-eGFP vector structure. The WT-a4 and V512L-a4 expressing plasmids were comstructed using the eukaryotic expression vector pcDNA3.1(+)

Table S1. Human vacuolar H^{+}-ATPase subunits, chromosomal localization and tissue distribution

Gene	Subunits	Chrosomal Localization	Size kDa	Tissue Localization	UniProt ID	NCBI Acession
V1						
ATP6V1A	V1 subunit A	3q13.31	70 kDa	ubiquitous	P38606	NM_001690
ATP6V1B1	V1 subunit B1	2p13	58 kDa	Kidney, inner ear, epididymis	P15313	NM_001692
ATP6V1B2	V1 subunit B2	8p22-p21	58 kDa	Ubiquitous	P21281	NM_001693
ATP6V1C1	V1 subunit C1	8p22-q21.3	42 kDa	Ubiquitous	P21283	NM_001695
ATP6V1C2	V1 subunit C2	2p25.1	42 kDa	Kidney, placenta	Q8NEY4	NM_144583
ATP6V1D	V1 subunit D	14q23-q24.2	34 kDa	Ubiquitous	Q9Y5K8	NM_015994
ATP6V1E1	V1 subunit E1	22q11.21	31 kDa	Ubiquitous	P36543	NM_001696
ATP6V1E2	V1 subunit E2	2p21	31 kDa	Testis	Q96A05	NM_080653
ATP6V1F	V1 subunit F	7q32.1	14 kDa	Ubiquitous	Q16864	NM_004231
ATP6V1G1	V1 subunit G1	9q33.1	13 kDa	Ubiquitous	O75348	NM_004888
ATP6V1G2	V1 subunit G2	6p21.3	13 kDa	Brain	O95670	NM_138282
ATP6V1G3	V1 subunit G3	1q32.2	13 kDa	Kidney	Q96LB4	NM_133262
ATP6V1H	V1 subunit H	8p22-q22.3	50/57 kDa	Ubiquitous	Q9UI12	NM_015941
V0						
ATP6V0A1	V0 subunit a1	17q21	116 kDa	Ubiquitous	Q93050	NM_005177
ATP6V0A2	V0 subunit a2	12q24.31	116 kDa	Ubiquitous	Q9Y487	NM_012463
ATP6V0A3	V0 subunit a3	11q13.4-13.5	116 kDa	Osteoclasts	Q13488	NM_006019
ATP6V0A4	V0 subunit a4	7q33-34	116 kDa	Kidney, inner ear, epididymis	Q9HBG4	NM_130841
ATP6V0B	V0 subunit c"	1p32.3	21 kDa	Ubiquitous	Q99437	NM_004047
ATP6V0C	V0 subunit c	16p13.3	16 kDa	Ubiquitous	P27449	NM_001694
ATP6V0D1	V0 subunit d1	16q22.1	38 kDa	Ubiquitous	P61421	NM_004691
ATP6V0D2	V0 subunit d2	8q21.13	38 kDa	Kidney,osteoclast, lung	Q8N8Y2	NM_152565
ATP6V0E1	V0 subunit e1	5q35.1	9 kDa	Ubiquitous	O15342	NM_003945
ATP6V0E2	V0 subunit e2	7q36.1	9 kDa	Ubiquitous	Q8NHE4	NM_001367789
Transport accessory protein						
ATP6AP1	Interacting protein1	Xq28	45 kDa	Ubiquitous	Q15904	NM_001183
ATP6AP2	Interacting protein2	Xq21	9 kDa	Ubiquitous	O75787	NM_005765

Table S2. The key clinical information of the patient

Examinations	Value	Reference
Aldosterone（pg/ml）	342.92	10.00-160.00
AngiotensinII(pg/ml)	75.00	25.00-129.00
Renin（pg/ml）	123.15	4.00-24.00
ARR	2.78	＜38.00
Dopamine(pmol/L)	994.3	＜195.7
Adrenaline(pmol/L)	126.2	LP＜605.4, SP＜769.0
Normeta nephrines(pmol/L)	3031.2	LP:414.0-4435.5, SP:1182.8-10054.0
Abbreviations: : ARR, aldosterone/reninratio;LP, lying position; SP, standing position; .		

Table S3. Laboratory examinations of the patient and the patient's father

**Laboratory examinations**	**Results**
**Patient**	
computed tomography (CT) of the kidneys and adrenal glands	No thickening of the bilateral adrenal glands was observed, and the renal CT showed no abnormalities.
Renal ultrasound of bilateral kidneys and renal arteries	The left kidney measures 96×40mm, and the right kidney measures 98×40mm. Both kidneys are normal in size and shape, with distinct corticomedullary demarcation and no separation of the collecting system. The main renal arteries are clearly visualized and follow a natural course.
Electrocardiogram	Sinus rhythm, left ventricular hypertrophy, and T wave abnormalities.
Echocardiography	The left atrium and left ventricle are enlarged, with reduced wall motion. Mild to moderate mitral regurgitation is present. Mild pulmonary hypertension is noted, along with a small pericardial effusion. Ejection Fraction (EF): 46%.
**Patient's father**	
Renal ultrasound of bilateral kidneys ,adrenal glands and renal arteries	The left kidney measures 111×55 mm, and the right kidney measures 112×58mm. An 11*12 mm fluid-filled anechoic area is observed in the right kidney, suggesting the presence of a right renal cyst. Both kidneys are normal in size, with distinct corticomedullary demarcation and no separation of the collecting system. The main renal arteries are clearly visualized and follow a natural course.
Electrocardiogram	Sinus rhythm and T wave abnormalities.
Echocardiography	The left atrium is mildly enlarged, measuring 46 mm in transverse diameter and 53 mm in longitudinal diameter. The remaining cardiac chambers, major vessels, and valvular structures are within normal limits without evidence of abnormalities.

Table S4. Prediction results of the pathogenicity of the ATP6V0A4 p.V512L mutation

Prediction tools	Pathogenicity
SIFT	Neutral
MutPred2	Likely pathogenic
PANTHER	Probably damaging
PolyPhen2	Probably Damaging
Mutation taster	Disease causing

Note: SIFT (http://sift.jcvi.org)

MutPred2(http://mutpred.mutdb.org/)

PANTHER(http://www.panther.org)

PolyPhen-2(http://genetics.bwh.harvard.edu/pph2/)

Mutation taster(http://www.mutationtaster.org/)

Table S5. CADD scores

**CADD assessment of impact of ATP6V0A4 p.V512L variant**							
	**DNA coordinates**				**CADD scores**		
Variant	CADD	Chrom.	Coord	Codon change	raw	PHRED	Significance
Val512Leu		7	138739578	Gtg/Ctg	3.55	24.30	Possibly deleterious
Val512Leu		7	138739578	Gtg/Ctg	3.52	24.30	Possibly deleterious

Table S6. Quality evaluation of the structural models for the WT-a4 and V512L-a4 subunits.

models	Procheck				ERRAT	Verify3D
	Core	allow	Generally, allow	disallow		
WT	93.1%	6.4%	0.3%	0.3%	96.59	46.19%
V512L	92.3%	6.8%	0.6%	0.3%	96.27	42.87%

Note: According to the Ramachandran plot analysis via the Procheck web server, more than 90% of the amino acid residues fall within the core and generally allowed regions, indicating high-quality structures for both proteins. ERRAT values above 95% suggest high-resolution protein models, while Verify3D scores of 42% and 46% indicate moderate structural quality for both proteins.

Table S7. Secondary structure percentages of the WT-a4 and V512L-a4 subunits

Mutation	structure	Coil	β-Sheet	β-Bridge	Bend	Turn	A-Helix	5-Helix	3-Helix
V512L	0.76	0.14	0.07	0.01	0.06	0.12	0.56	0.00	0.04
WT	0.76	0.14	0.07	0.01	0.06	0.12	0.56	0.00	0.04

*** Structure = β-sheet + β-bridge + Turn+ A-helix.**

Note: The changes in secondary structure during MD simulations were calculated using DSSP software. The percentage of each secondary structure in WT-a4 and V512L-a4 subunits was assessed, and no significant changes were observed in the structural profile of the V512L-a4 compared to the WT-a4 subunits.

Table S8. Docking scores and binding free energy values for V512L-a4 with selected ligand compounds.

Compounds	molecular formulas	Structure	Docking score (kcal/mol)	MM/GBSA ΔTotal(kcal/mol)
Relamorelin	$C_{43}H_{50}N_8O_5S$	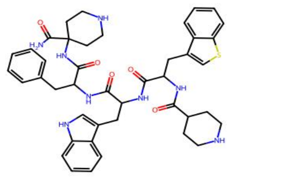	-10.226<br>（vina-8.369 ）	-70.72（gromacs-58.54）
Forsythiaside A	$C_{29}H_{36}O_{15}$	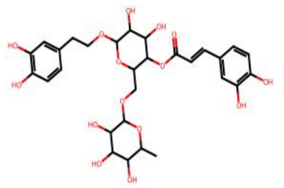	-10.035<br>（vina-8.315 ）	-79.14（-43.96）
PAR-1AC	$C_{35}H_{58}N_{10}O_9.C_2H_4O_2$	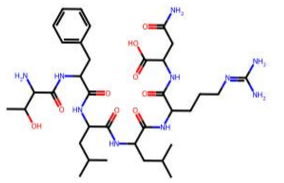	-11.396<br>（vina-6.201 ）	-69.91（-52.18）
F359-0497	$C_{26}H_{22}FN_5O_3S$	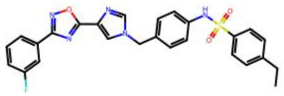	-10.235	-52.82
F351-0364	$C_{25}H_{26}FN_3O_2S$	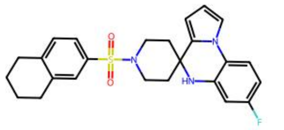	-10.743	-51.05

Table S9. Binding free energy (in kcal/mol) and its components for the docking of five potential inhibitor compounds with V512L-a4 subunit , as calculated using MM/GBSA.

Contribution	Relamorelin	Forsythiaside A	PAR-1 AC	F359-0497	F351-0364
ΔE_{vdw}	-44.70	-69.10	-71.70	-64.78	-55.33
ΔE_{ele}	-87.72	-34.96	-81.89	-8.02	-20.48
ΔG_{gas}	-132.42	-104.06	-153.58	-72.80	-75.82
ΔG_{solv}	73.87	60.11	101.41	19.98	24.77
ΔG_{Total}	-58.54	-43.96	-52.18	-52.82	-51.05

Table S10. The primers of WT-a4 and V512L-a4 plasmid construction.

Primers	Sequence
**ATP6V0A4-Forward**	CTGCTGGAGTTCGTGACCGCCGCCGGGATCACTCACGGCATGGACGAGCTGTACAAGGGTACC
**ATP6V0A4-Reverse**	GCGGGTTTAAACGGGCCCTCTAGACTCGAGCGGCCGCCACTGTGCTGGATATCTGCAGAATTC
