# Supplementary material for: A gain-of-function mutation in ATP6V0A4 drives primary distal renal tubular alkalosis with enhanced V-ATPase activity
Source: J Clin Invest. 2025 Apr 29;135(13):e188807. doi: 10.1172/JCI188807 (PMC12208546; doi:10.1172/JCI188807)

A gain-of-function mutation in ATP6V0A4 drives primary distal renal tubular alkalosis with enhanced V-ATPase activity

Unedited blot and gel images

- **Figure 3**

- **Figure3-B 293T_A4**

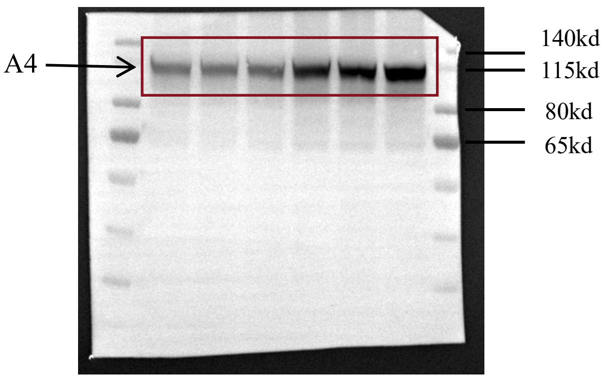

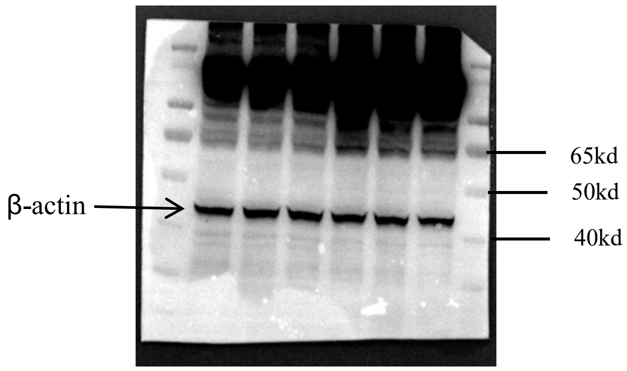

- **Figure3-B M1_A4**

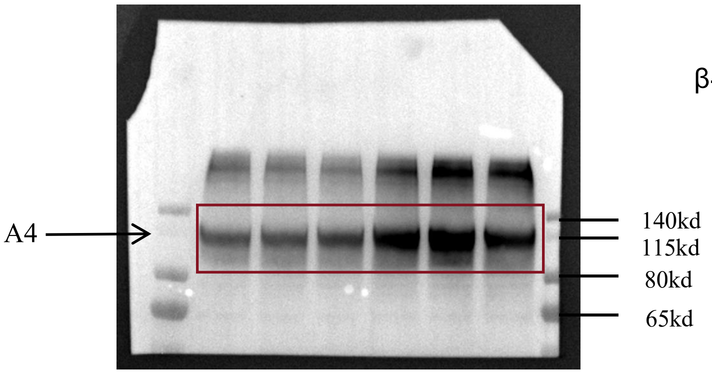

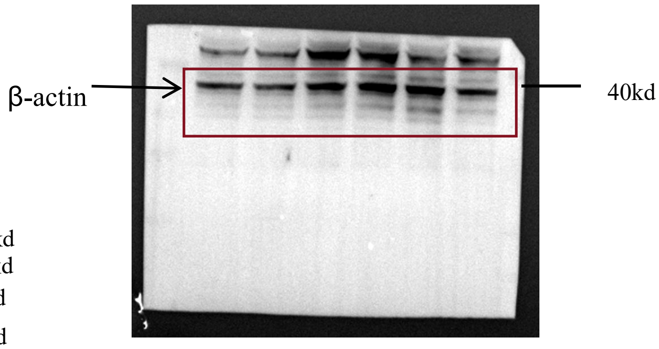

- **Figure3-G_WT-A4**

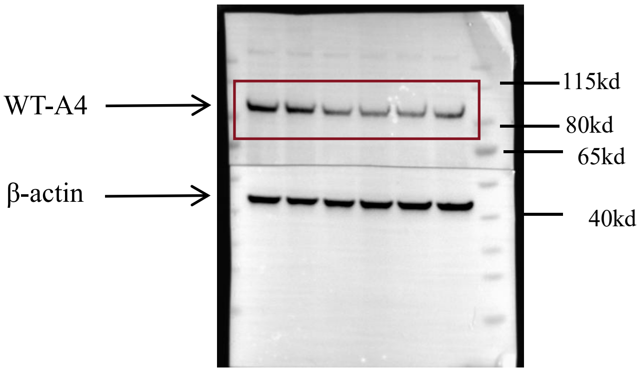

- **Figure3-G_V512L-A4**

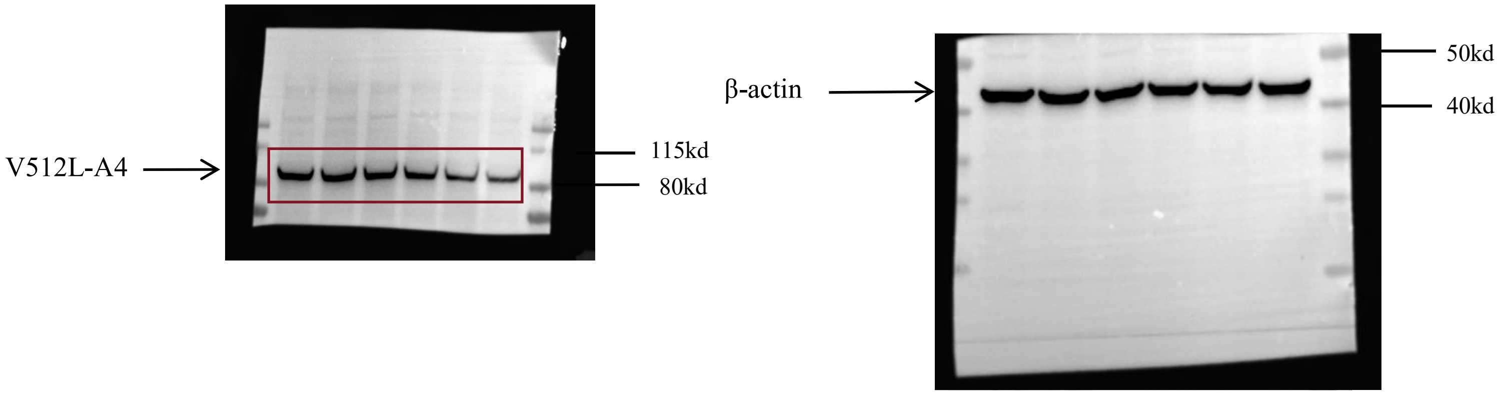

- **Figure 5**

- **Figure 5-C_P62**

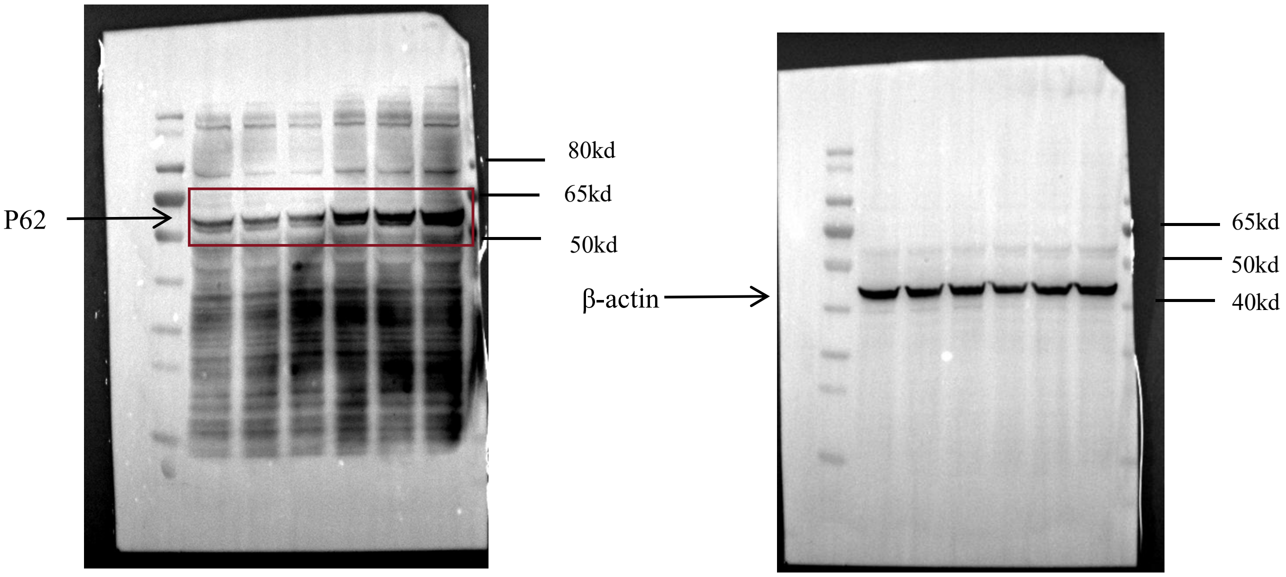

- **Figure 5-C_LC3**

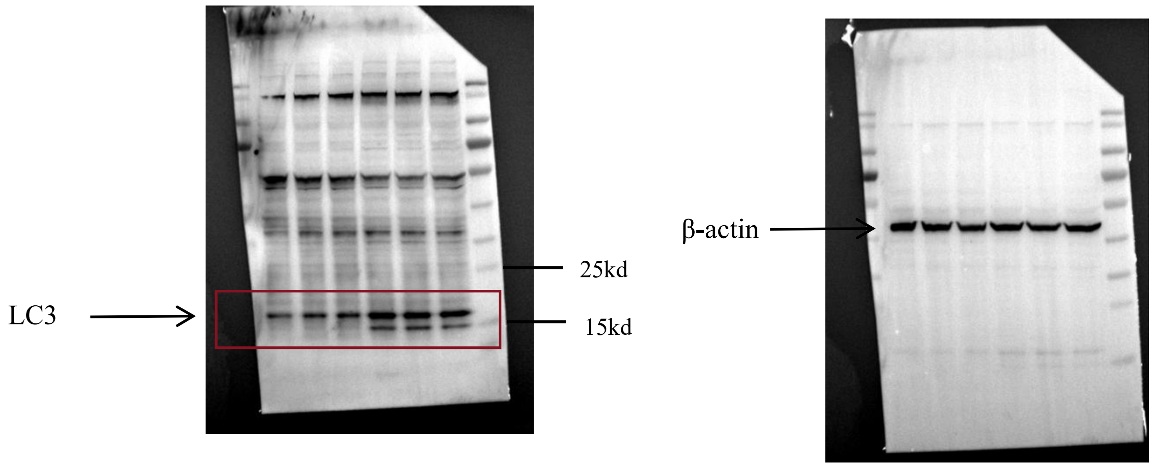

- **Figure 5-J_Bax**

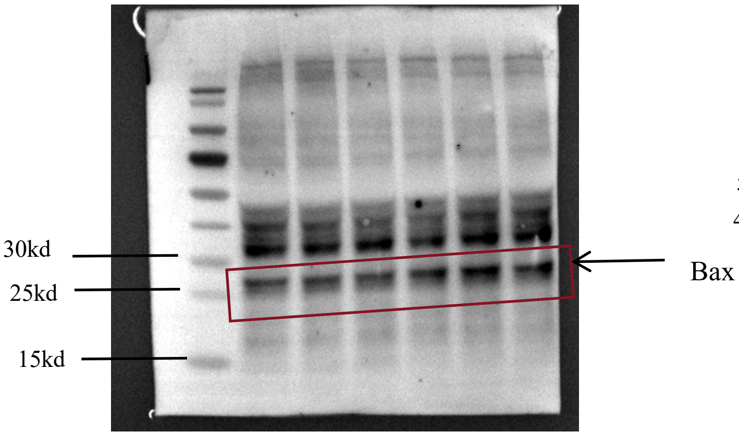

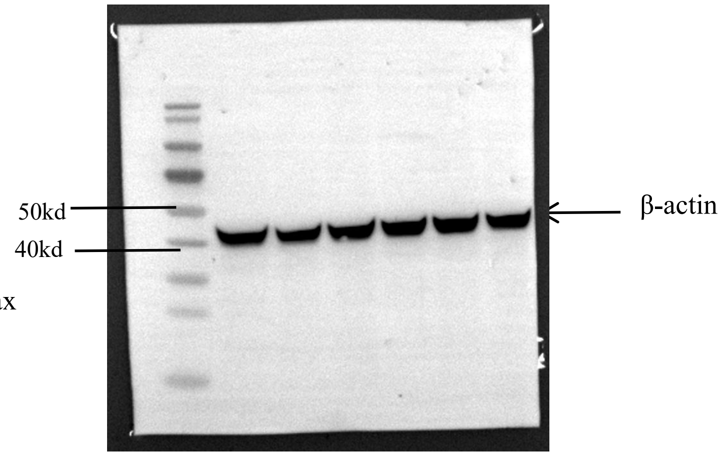

- **Figure 5-J_Cle-cas3**
- **Figure 5-J_Bcl-2**

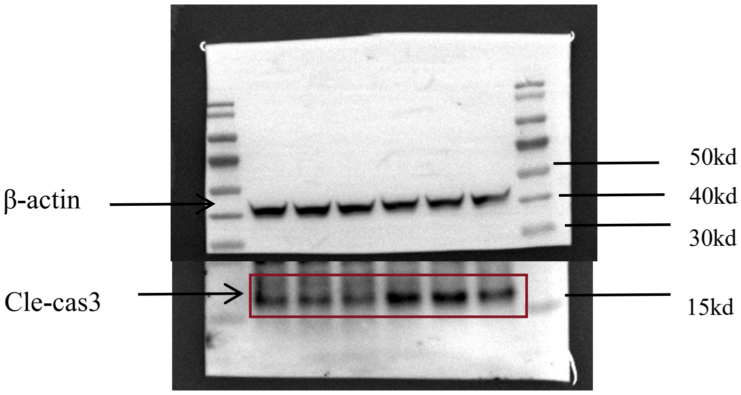

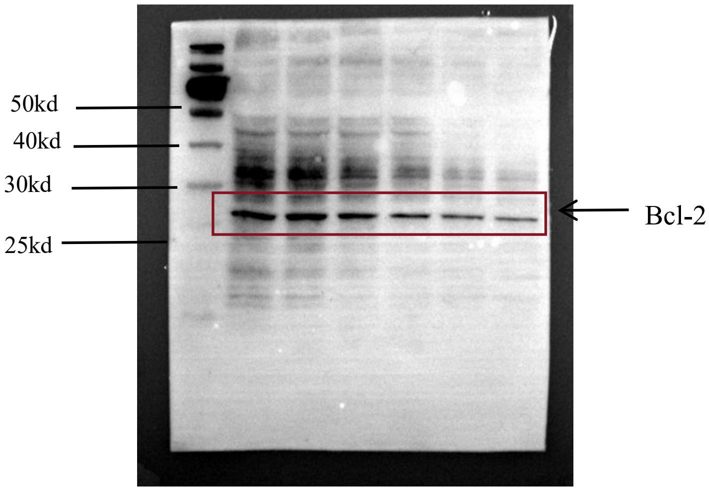

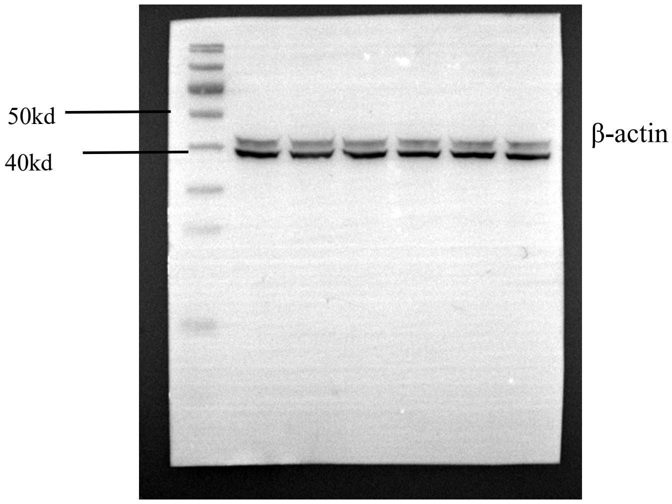

◆ **Figure 7**

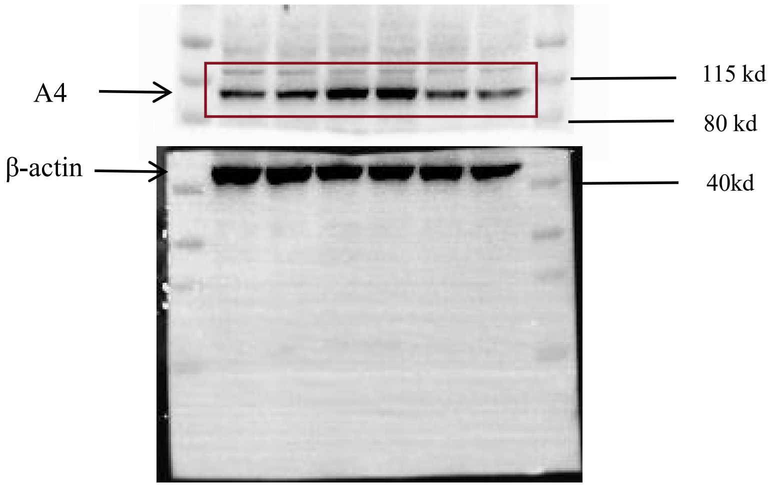

- **Figure 8**
- **P62+LC3**

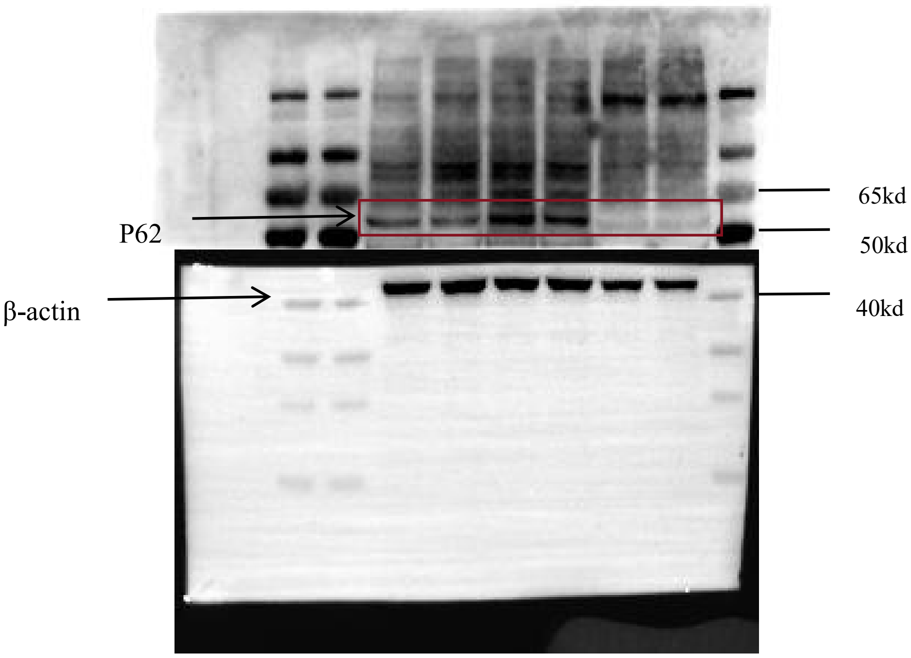

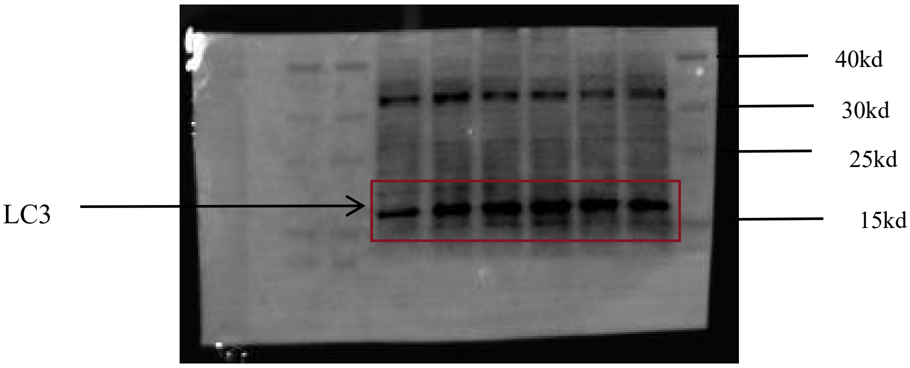

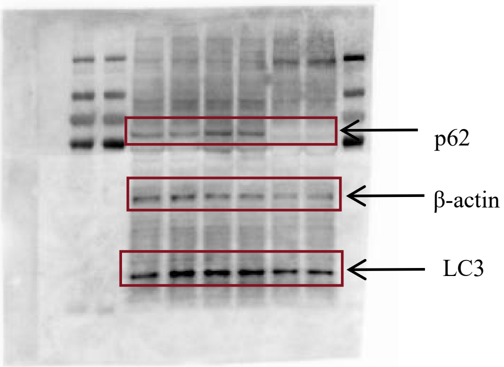

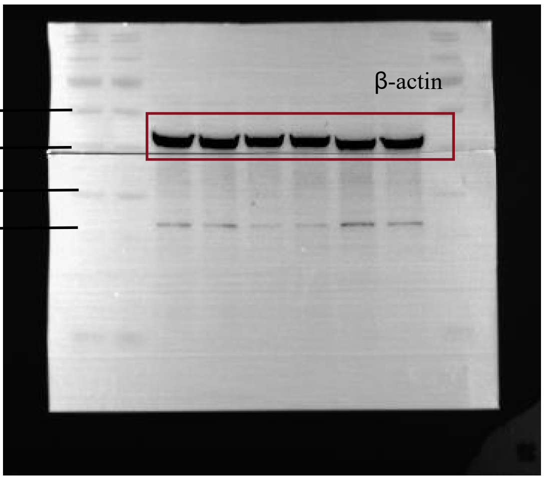
β-actin
50kd
40kd
30kd
25kd

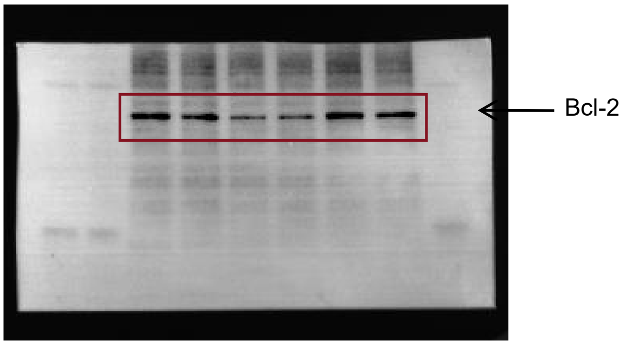
Bcl-2

- **Bcl-2**

- **Bax**

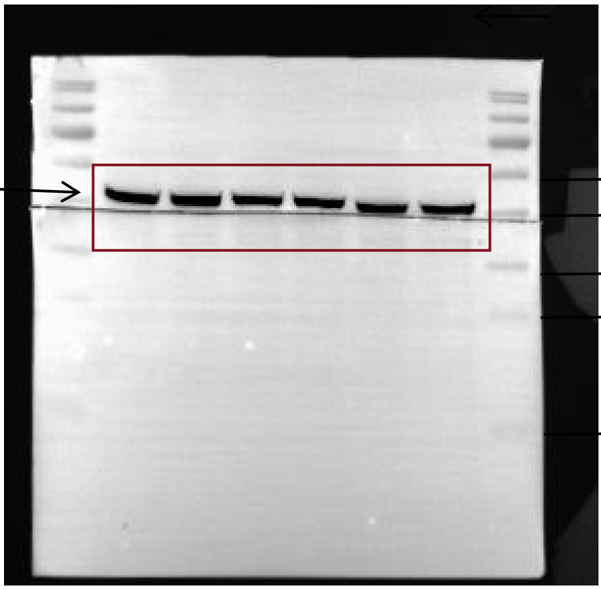
β-actin
50kd
40kd
30kd
25kd
15kd

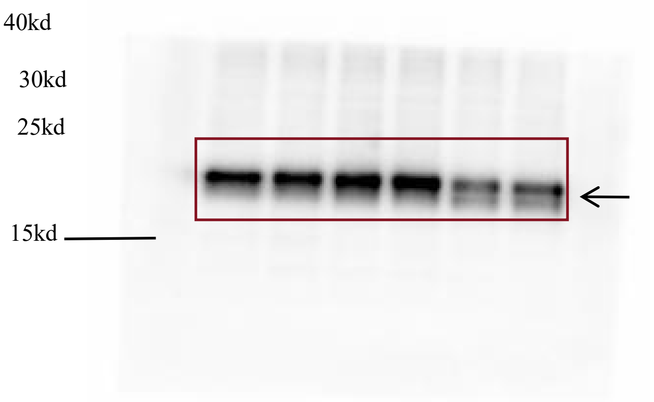
Bax

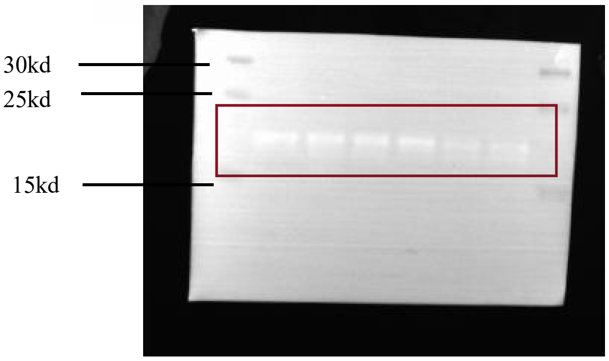
30kd
25kd
15kd
Bax

- **Cle-casp3**

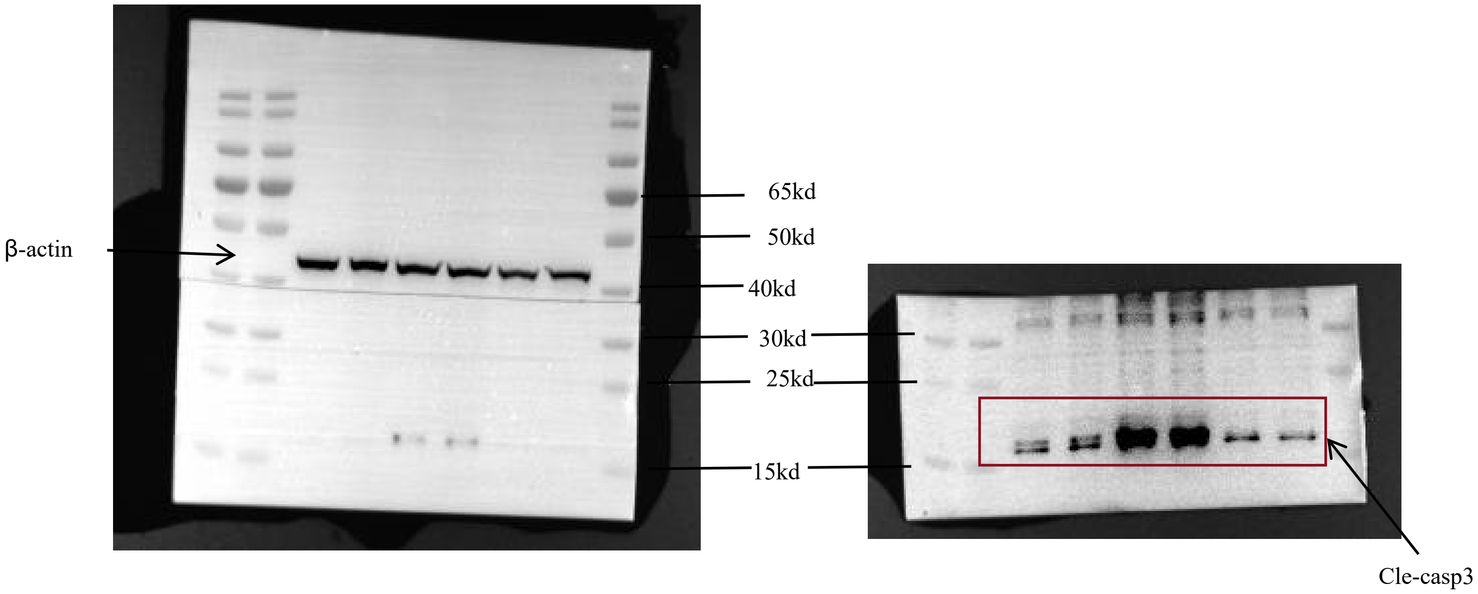

Supplement_Fig S9_A

A4

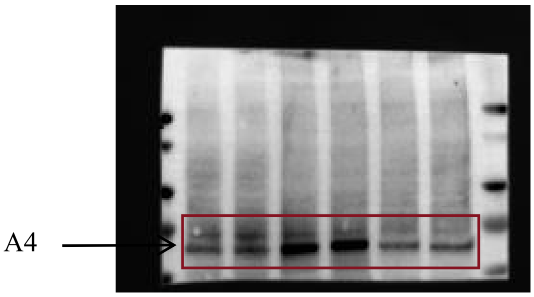

β-actin

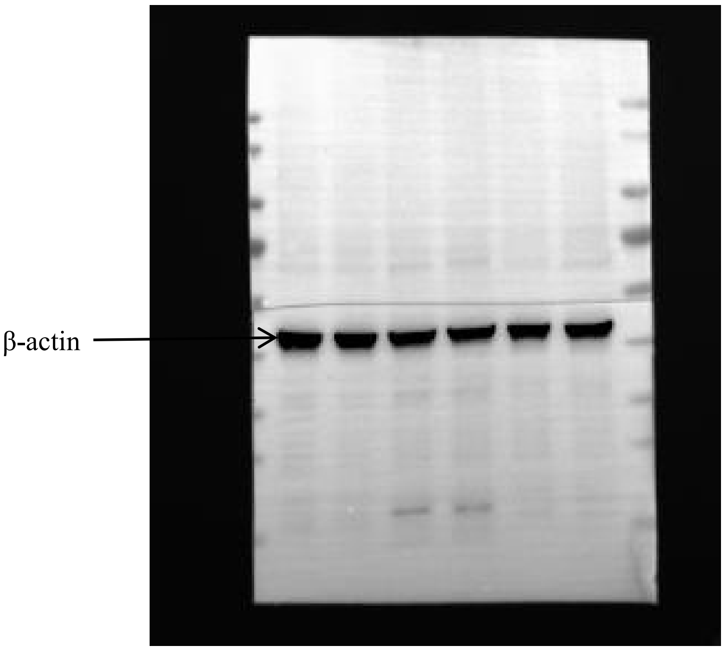

Supplement: Unedited blot and gel images [file jci-135-188807-s245.pdf]
